# Individual variation in role construal predicts responses to third-party biases in hiring contexts

**DOI:** 10.1371/journal.pone.0244393

**Published:** 2021-02-03

**Authors:** Andrea C. Vial, Janine Bosak, Patrick C. Flood, John F. Dovidio

**Affiliations:** 1 Department of Psychology, Division of Science, New York University Abu Dhabi, Abu Dhabi, United Arab Emirates; 2 DCU Business School, Dublin City University, Dublin, Ireland; 3 Department of Psychology, Yale University, New Haven, CT, United States of America; Pontificia Universidade Catolica do Rio Grande do Sul, BRAZIL

## Abstract

We theorize that individuals’ pre-existing beliefs about the hiring manager role (role construal) are associated with their tendency to condone bias accommodation in hiring contexts, in which a person aligns hiring decisions with the perceived biases of others. In two studies, we focus on human resources (HR) professionals’ endorsement of the role demand to prioritize candidate fit with others (e.g., supervisor) when making hiring decisions. Study 1 examined bias accommodation from a vicarious perspective, revealing that role demand endorsement is positively associated with viewing it as acceptable and common for *another* hiring manager to accommodate third-party bias against women. Study 2 examined bias accommodation experimentally from an actor’s perspective, showing lower preference for and selection of a female (vs. male) job candidate in the presence of cues to third-party bias against women, but only when role demand endorsement is relatively high. HR professionals in both studies indicated that third-party bias influences in hiring are relatively common. Responses in Study 2 provide preliminary evidence that the phenomenon of third-party bias accommodation might be relevant in the context of employment discrimination based on group characteristics other than gender (e.g., race/ethnicity, age). We discuss the practical implications of our findings for hiring professionals and for organizations seeking to increase diversity in their workforce.

## Introduction

Organizations frequently outsource staff recruitment efforts to external providers [1, 2]. Outsourcing recruitment processes can be beneficial, increasing efficiency and reducing overall costs associated with recruitment [3]. Outsourcing the hiring process can also provide access to expertise and best practices in hiring [4, 5], and allow organizations to reach higher-quality pools of candidates [4, 6]; cf. [1]. However, subtle biases may compromise the goal of hiring top talent even through outsourcing [7, 8]. The focus of the current investigation is on understanding how hiring professionals, when making hiring decisions on behalf of another organization, may accommodate the perceived biases of existing organizational members due to the manner in which they construe their professional role. Bias represents an unfair orientation toward a group and its members that can involve stereotypes (beliefs about the characteristics of a group and its members), prejudice (a group-based attitude), and/or discrimination (behavior that unfairly disadvantages a group and its members) [9].

We theorize and examine how and why hiring managers and professionals respond to the social biases of *other people* in the context of hiring decisions, and to the possibility of spreading those biases. Individuals operate in accordance with what they see as the demands of their formal role [10, 11], and when making recruitment decisions on behalf of others, this role construal can lead professionals to align their hiring decisions with the perceived biases (prejudice, stereotyping, and/or discriminatory actions) of relevant third parties. This bias accommodation process has been labelled the “third-party prejudice effect” and has only very recently been established in psychological experiments [12, 13]. Crucially, in the current work, we propose that the influence of third-party bias depends in part on the way that individuals construe the hiring manager role—a construal that is likely shaped by professionals’ training, personal histories and attitudes, and belief systems. Thus, we examined the responses of experienced hiring professionals, for the first time, to our knowledge, in two studies.

Given substantial evidence that hiring discrimination contributes to persistent gender segregation in the labor market [14], we focus our research on hiring discrimination as a result of the inferred gender biases of a relevant third party. Throughout this investigation, we refer to the consequence of accommodating third-party bias on employment decisions as “hiring discrimination,” defined as excluding candidates based on group membership regardless of their qualifications, in this case, due to the perceived gender bias of a third party [12, 13].

Theoretically, we offer insight into the prevalence of the third-party bias effect and why it occurs. We focus on a mechanism, individual differences in role construal—specifically, endorsement of a role demand for hiring managers to prioritize candidate fit with others—that explains why people might condone bias accommodation in hiring. We also introduce a novel perspective into this line of research—professional observers’ reactions to others who accommodate third-party bias—that illuminates how endorsing a role demand to maximize candidate fit with others is associated with finding it acceptable and normative for third-party bias against women to be accommodated. Empirically, we investigate the association between individual variation in role construal and professionals’ (i) responses to others who accommodate third-party bias in hiring; and (ii) own tendency to accommodate the biases of relevant third parties in hiring. The samples for this research included hiring professionals in the Republic of Ireland (Study 1) and in the United States (Study 2).

### Theoretical framework

We approach bias accommodation within the theoretical framework of role theory [15, 16], and we test how professional hiring managers’ role construal (i.e., their understanding of the formal demands of being in a hiring role) may influence their views about bias accommodation in hiring contexts. Whereas past work with nonprofessional samples manipulated the contextual strength of role-related concerns to examine the influence of role demands on bias accommodation [12, 13], the current investigation is the first to examine how pre-existing individual differences in the endorsement of specific role duties—the beliefs about their role that hiring professionals bring to the table—may promote hiring discrimination as a result of bias accommodation. In doing this, we contribute to a more thorough understanding of the third-party bias effect by testing individual differences rather than situational factors that contribute to it. Moreover, we combine this role-based framework with theory on actor-observer discrepancies [17, 18] to examine not only trained professionals’ own vulnerability to accommodating third-party bias as a function of their role construal, but also how they respond to others who accommodate bias. Thus, the present research contributes to the further development of role theory by testing how role construal shapes judgments of the in-role behaviors of others, an underexamined question with important practical implications in the context of bias accommodation.

#### Accommodation of others’ biases in hiring contexts

Recent empirical research reveals how individuals making hiring decisions on behalf of a third party may accommodate third-party biases against women. In a series of experiments with nonprofessional samples, Vial and colleagues [12, 13] randomly assigned college students, community volunteers, and adults on Amazon Mechanical Turk to hypothetical roles in charge of hiring decisions (e.g., hiring manager). Across studies, participants tended to make discriminatory hiring decisions against female job candidates when they had reason to suspect that a relevant third party (someone with whom the new hire would work closely) was biased against women (the “third-party prejudice effect”). Qualitative evidence suggests that third-party sexism may operate in significant ways outside the laboratory as well. Fernandez-Mateo and King [8], who interviewed staffing consultants in charge of screening job candidates for a temporary employment firm in the United Kingdom, found that consultants tried to anticipate clients’ preferences for male and female candidates (a phenomenon labeled “anticipatory gender sorting”). The consultants assumed that clients would be less interested in hiring women for top-paying jobs; therefore, they tended to disproportionately sort female candidates into low-paying jobs and male applicants into high-paying positions, despite of similar qualifications.

In the current investigation, we integrate these different streams of research [8, 12, 13] and examine the third-party bias effect in trained hiring professionals, making a number of contributions to advance theory on bias accommodation in hiring contexts. Hiring professionals typically receive training to curtail the impact of bias in the candidate selection process [19]; thus, they might be less personally susceptible to bias accommodation and condone others’ bias accommodation less than would people outside the profession. However, we theorize that trained professionals are amenable to accommodating others’ biases to the extent that doing so aligns with the way that they construe the hiring manager role. To test this idea, we examine whether role construal—specifically, endorsing a role duty to consider the preferences of existing organizational members and to prioritize a new hire’s fit with those individuals—might predict hiring professionals’ responses to third-party bias accommodation.

#### Individual variation in role construal

Role theory [15, 16] posits that people behave in ways that are consistent with the priorities of the formal role they occupy in a given moment [10, 20], especially when the role is characterized by strong, unambiguous demands and duties [21]. Consistent with this theory, past studies showed that situational variation in the strength of role demands (e.g., having more vs. less at stake for the organization) moderated responses to third-party bias [12, 13], such that lay respondents only accommodated others’ biases when the potential organizational consequences of not doing so were very dire (i.e., when role demands were strong vs. weak). In the current investigation, we build on these prior findings on situational role factors by examining whether individual differences in professionals’ construal of the hiring manager role predict their responses to bias accommodation. Indeed, there is individual variability in the way that people experience the roles they occupy, which is in turn associated with differences in role behavior [11, 22–24]. From this perspective, we propose that whether individuals in roles that involve hiring decisions would condone bias accommodation might depend on how they construe their role.

Specifically, beyond identifying top talent, those who evaluate job candidates often believe that they must consider the compatibility or “fit” of prospective candidates with the organizations or groups in which they would work [25–27]. Prioritizing candidate fit with others is reasonable because it can be beneficial for organizations: Strong fit is associated with positive work attitudes and performance [28, 29], whereas weak fit is linked with conflict, turnover, and poor performance [30]. We investigate individual differences in endorsement of this role demand to prioritize candidate fit with others in hiring, testing whether hiring professionals who see maximizing candidate fit with others as a more central role priority would be more inclined to exclude certain job candidates, regardless of their qualifications, due to third-party bias.

#### Condoning others’ accommodation of bias

The way that individuals construe the demands of specific roles might set behavioral standards for role occupants in general. However, to date, no research that we know of has examined the way that role construal shapes individuals’ judgments of the in-role behaviors of *another* person. Nevertheless, understanding whether hiring managers would condone bias accommodation when *other professionals* do it is of both theoretical and practical importance. For example, in the commonly recommended practice of multi-person hiring panels, for which two or more raters compare their assessments of a job applicant [31, 32], how professionals’ respond to each other’s tendency to accommodate bias could be consequential.

Research on actor-observer discrepancies [17] suggests that, even if they are personally susceptible to accommodating third-party bias, hiring managers might disapprove of others’ accommodation decisions. Given that observers have direct knowledge of their own intentions but usually possess only incomplete knowledge of others’ intentions, they often attribute others’ behaviors to internal dispositions rather than external factors [17], particularly for negative events [18] such as discrimination. Thus, even though they themselves might personally accommodate third-party bias to meet formal role demands, it is possible that hiring professionals might see this behavior as a signal of personal bias when *others* do it [33], and therefore disapprove of it. If so, hiring professionals might keep each other in check, potentially containing the spread of bias.

However, unlike the kinds of behaviors for which actor-observer differences in behavioral attribution are common (e.g., donating to charity) [17, 18, 34], it is possible that such discrepancies may be minimal when judging in-role behaviors because formal roles provide a more or less consensual blueprint or script for role occupants [10, 15]. Knowledge of the role and associated duties might provide a key piece of information for observers (filling the gap of the unobservable intention) and provide a rationale to explain observed decisions that appeal to the structural constraints imposed by the formal role rather than internal dispositions such as personal bias [33]. Thus, we propose that endorsement of the role demand to maximize candidate fit with others may lead individuals responsible for hiring to condone bias accommodation as more or less required of any role occupant.

### Overview of studies and hypotheses

A primary goal of the current research is to investigate the underexamined relationship between variability in role construal and responses to bias accommodation in human resources (HR) professionals, who have extensive knowledge of hiring practices and may develop more enduring role orientations through their relevant work experiences. Whereas situationally varying the role demands to be prioritized in a given context has been shown to influence bias accommodation [12], such manipulations are subject to experimental demands and may not represent the dynamics of more naturalistic decision making, or the effects of a more stable role construal, which we examined in the current studies. Moreover, in addition to studying the effects of role construal on their own behavior, we extend earlier work by investigating how role construal predicts HR professionals’ responses to others’ decisions to accommodate third-party bias. If behavior that is perceived to be “in-role”—that is, associated with central role demands and duties—is generally favored [22, 23], then HR professionals’ endorsement of a role demand to maximize candidate fit with others [25] may lead them to condone bias accommodation as more or less required of any role occupant.

Based on this reasoning, we hypothesize that higher endorsement of the role demand to maximize candidate fit with others is associated with responses that promote bias accommodation, and we test this hypothesis from two different perspectives (Hypothesis 1a, 1b, and 1c). First, in Study 1 we investigate the relationship between endorsing the role priority to maximize candidate fit with others and finding it (a) acceptable and (b) descriptively normative (i.e., common) for a hiring manager to accommodate third-party bias against women:

**Hypotheses 1a-b.** Endorsement of the role demand to prioritize candidate fit with others will be associated with finding it acceptable (H1a) and normative (H1b) for someone to accommodate third-party bias against women.

By taking a vicarious perspective on the third-party bias effect and investigating how role construal shapes judgments of the in-role behaviors of others, we extend past work [12, 13] that focused solely on the actor’s perspective in a novel way that can contribute to the further development of role theory.

Second, in Study 2 we examine how endorsement of the role priority to maximize candidate fit with others may influence HR professionals’ own tendency to accommodate third-party bias against women. The experimental approach in Study 2 can provide causal evidence to support our claim that inferences about the biases of others can sway the decisions of hiring managers and professionals in ways that place female job candidates at a disadvantage, much like the nonprofessional samples tested by Vial et al. [12, 13], complementing and extending past field investigations on anticipatory gender sorting [8]. Moreover, Study 2 builds on past experimental work that examined the third-party bias effect as a function of situational variation in the strength of role demands 12, 13] but advances the same by testing how individual differences in role construal might moderate the effect. We test the following:

**Hypothesis 1c.** High endorsement of the role demand to prioritize candidate fit with others will be associated with lower selection of a female job candidate when there are cues to gender bias in a relevant third party.

Studies 1 and 2 are designed to extend previous work on the third-party bias effect by probing the relationship between responses to bias accommodation and three role-related concerns that may arise upon learning of another’s bias in hiring contexts. Two of these concerns—an *interpersonal concern* that the biased third party would not get along well with a female hire and thus disrupt cohesiveness, and a *task-focused concern* that a female hire could not perform well in this context of bias—were implicated in past research with lay samples [12, 13]. In Study 1 of the present research, we examine a third possible concern that may be particularly relevant to actual HR professionals (compared to nonprofessional participants in the previous research by Vial et al., [12, 13])—a *professional concern* by hiring managers that failure to accommodate the wishes of relevant parties (including a biased person) would jeopardize their own organizational standing and reputation. For the three sets of role-related concerns (interpersonal, task-focused, and professional), we hypothesize positive relationships with responses that promote bias accommodation (relating to one’s own behavior and the attributions made to others’ behavior). We tested the following in Study 1:

**Hypotheses 2a-b.** Interpersonal, task-focused, and professional concerns about a female candidate’s fit with others will correlate positively with finding it acceptable (H2a) and normative (H2b) for someone to accommodate gender-based third-party bias.

In Study 2, we expect role-related concerns to underlie hiring professional’s decisions in contexts of third-party bias against women:

**Hypotheses 2c-d.** Interpersonal and task-focused concerns about a female candidate’s fit with others will be higher when there are cues to gender bias in a relevant third party (H2c), and will mediate the effect of third-party bias cues on hiring decisions (H2d).

In addition to these central hypotheses, we examine the effects of respondent gender and participants’ egalitarianism on their reactions to the accommodation of third-party bias against women in Study 1. First, we expect that responses might vary with participant gender, based on a large body of research showing that people are more likely to perceive and respond negatively to bias against members of their own group than against members of another group [35]. Specifically, we expect that female participants might condemn bias accommodation against women (a social in-group) more strongly than would male participants (for whom women are an out-group). We hypothesize:

**Hypothesis 3.** Female (vs. male) respondents will find it less acceptable for someone to accommodate gender-based third-party bias against women.

Moreover, we expect that the extent to which participants condone or condemn bias accommodation might vary with their own beliefs about gender egalitarianism. Past work shows that more egalitarian mindsets have an impact on social decisions, reducing bias and discrimination [36, 37]. Thus, we hypothesize a relationship between participant’s egalitarian beliefs and their responses to gender-based bias accommodation generally:

**Hypothesis 4.** Endorsing more gender egalitarian ideals will correlate negatively with finding it acceptable for someone to accommodate gender-based third-party bias.

In the current studies, we examined—for the first time, to our knowledge—the potential interplay between individual level-factors and dispositional role construal on responses to bias accommodation. Past investigations provided some evidence that respondent gender and ideology do not moderate the bias accommodation effect when situational role demands are strong [12, 13]. However, it remains unclear whether or how an individuals’ group identity or egalitarianism might interact with a more stable and enduring role construal. For those whose role construal involves a strong priority for hiring managers to maximize candidate fit with others [25], the influence of ingroup preferences and egalitarian beliefs on responses to bias accommodation would be expected to be minimal [10]. To explore these possibilities, we examine whether endorsement of role demands moderates the effects of egalitarianism (Study 1) and respondent gender (Studies 1 and 2) on reactions to bias accommodation. We do not advance a strong prediction for these interaction effects given mixed findings on situational role demands [12, 13].

Finally, as a way to complement the insights of past field investigations on anticipatory gender sorting [8] and to further integrate this perspective with research on the third-party bias effect [12, 13], we seek to document hiring professionals’ personal experience with third-party bias accommodation and their perception of the prevalence of explicit client requests to discriminate based on demographic characteristics as well as the prevalence of inferred client biases. In Study 2, we also asked HR professionals in the U.S. to indicate which social groups were the targets of explicit demands and inferences, including not only gender groups but also others (e.g., based on race/ethnicity). Thus, whereas our primary focus was on gender bias, these responses may provide valuable insights on the generalization of the third-party bias effect to a larger set of social groups and its relevance to understanding hiring discrimination more broadly.

## Study 1: Do hiring professionals condone the accommodation of bias?

Study 1 examined how acceptable and normative hiring professionals would find the behavior of *another* professional who accommodated third-party bias against women in a hiring context, and whether those judgements varied with role demand endorsement, testing Hypotheses 1a-b, 2a-b, 3, and 4. Drawing on a paradigm used in previous work on the third-party bias effect [12], participants read about a hiring manager who chose to hire a man over a similarly qualified woman due to a relevant third-party’s (the Chief Executive Officer’s) conservative gender views, and then completed a series of measures, including endorsement of a role demand for hiring managers to prioritize candidate fit with others.

We also asked participants about their own personal experience with third-party bias in organizational contexts to complement the insights of past field investigations on anticipatory gender sorting [8] and to further integrate this perspective with research on the third-party bias effect [12, 13].

### Materials and methods

Data collection for Study 1 and Study 2 was approved by the Institutional Review Board at Yale University. Verbatim materials, data, and analysis code for the two studies are available on the Open Science Framework (OSF) database (https://osf.io/xkn93). Unless otherwise noted, all analyses were performed with SPSS (version 27).

Participants in Ireland completed an online survey in exchange for the opportunity to win 1 of 5 vouchers worth 20 Euro. We recruited participants in five different ways simultaneously (see S1 Supplement in S1 File), including authors’ personal networks, partnerships with professional organizations, invitations to current students and alumni from an HR Master’s program, and chain referrals. Recruitment source did not impact results. Out of 320 participants, we excluded fifteen (4.7%) for inattention and eleven (3.4%) for indicating their answers to be jokes or random (see Procedure and Measures). Gender was missing for 77 participants. The final sample was *n* = 294 (mean age = 42.09, *SD* = 9.78; 72.4% female; 17.7% students; 91.2% Irish; 97.7% White). A sensitivity power analysis (see S1 Supplement in S1 File) indicated that our sample was sufficient to detect small-to-medium effects. Participants on average had 12.82 years of experience in HR (*SD* = 8.92). Among non-students, average experience in HR was slightly higher (*n* = 166; range: 0–40; *M* = 14.13, *SD* = 8.94). Most participants (77.6%) were involved in hiring efforts at the time of the study, and over the past year (86.1%), in which they filled or helped fill an average of 18 positions (*SD* = 15.76). Additional sample characteristics are reported in Table 1.

**Table 1:** Sample characteristics in Study 1 (Ireland) and Study 2 (United States).

	Study 1: Irish Sample, *n* (%)			Study 2: American Sample, *n* (%)		
	All^1^	Women	Men	All^1^	Women	Men
*Overall*	294 (100)	157 (53.4)	60 (20.4)	284 (100)	177 (62.3)	69 (24.3)
*Past industry experience*						
Technology	47 (16.0)	36 (22.9)	10 (16.7)	82 (28.9)	54 (30.5)	28 (40.6)
Banking/Financial services	60 (20.4)	46 (29.3)	13 (21.7)	80 (28.2)	55 (31.1)	24 (34.8)
Non-profit	46 (15.6)	35 (22.3)	11 (18.3)	77 (27.1)	57 (32.2)	20 (29.0)
Healthcare/social assistance	29 (9.9)	23 (14.6)	6 (10.0)	68 (23.9)	52 (29.4)	16 (23.2)
Transportation/warehousing	13 (4.4)	8 (5.1)	5 (8.3)	48 (16.9)	36 (20.3)	12 (17.4)
Education/academia	36 (12.2)	28 (17.8)	8 (13.3)	46 (16.2)	31 (17.5)	15 (21.7)
Hospitality/leisure	26 (8.8)	18 (11.5)	7 (11.7)	43 (15.1)	24 (13.6)	19 (27.5)
Mining/construction/ manufacturing	24 (8.2)	14 (8.9)	10 (16.7)	44 (15.5)	32 (18.1)	12 (17.4)
Government	39 (13.3)	26 (16.6)	13 (21.7)	37 (13.0)	24 (13.6)	13 (18.8)
Advertising/media/marketing/public relations	13 (4.4)	9 (5.7)	4 (6.7)	40 (14.1)	25 (14.1)	15 (21.7)
Trade (retail or wholesale)	30 (10.2)	20 (12.7)	10 (16.7)	37 (13.0)	24 (13.6)	13 (18.8)
Utilities	17 (5.8)	10 (6.4)	7 (11.7)	21 (7.4)	14 (7.9)	7 (10.1)
Agriculture	7 (2.4)	6 (3.8)	1 (1.7)	10 (3.5)	7 (4.0)	3 (4.3)
*Current employment status*						
Employed full time	188 (63.9)	128 (81.5)	56 (93.3)	226 (79.6)	161 (91.0)	64 (92.8)
Employed part time	10 (3.4)	9 (5.7)	0 (0.0)	7 (2.5)	7 (4.0)	0 (0.0)
Freelancer/consultant	13 (4.4)	10 (6.4)	3 (5.0)	10 (3.5)	7 (4.0)	3 (4.3)
Retired	0 (0.0)	0 (0.0)	0 (0.0)	3 (1.1)	1 (0.6)	2 (2.9)
Unemployed, looking for HR work	4 (1.4)	4 (2.5)	0 (0.0)	0 (0.0)	0 (0.0)	0 (0.0)
Unemployed, looking for non-HR work	1 (0.3)	1 (0.6)	0 (0.0)	0 (0.0)	0 (0.0)	0 (0.0)
Other*	6 (2.0)	5 (3.2)	1 (1.7)	1 (0.3)	1 (0.6)	0 (0.0)
Missing	72 (24.5)	0 (0.0)	0 (0.0)	37 (13.0)	0 (0.0)	0 (0.0)
*Current role^2^*						
HR role in a non-HR service company	131 (66.2)	97 (70.8)	32 (57.1)	171 (73.4)	139 (82.7)	31 (48.4)
HR service company	15 (7.6)	9 (6.6)	6 (10.7)	51 (21.9)	26 (15.5)	25 (39.1)
Non-HR role	45 (22.7)	28 (20.4)	15 (26.8)	7 (3.0)	1 (0.6)	6 (9.4)
Other**	6 (3.0)	3 (2.2)	3 (5.4)	4 (1.7)	2 (1.2)	2 (3.1)
Missing	1 (0.5)	0 (0.0)	0 (0.0)	0 (0.0)	0 (0.0)	0 (0.0)
*HR role rank^2^*						
Entry-level HR	3 (1.5)	1 (0.7)	2 (3.6)	2 (0.8)	2 (1.2)	0 (0.0)
Mid-level HR	33 (16.7)	26 (19.0)	6 (10.7)	74 (31.8)	58 (34.5)	15 (23.4)
Upper-level HR	64 (32.3)	49 (35.8)	15 (26.8)	64 (27.5)	39 (23.2)	25 (39.1)
Head of HR	41 (20.7)	27 (19.7)	13 (23.2)	60 (25.7)	50 (29.8)	10 (15.6)
HR functions	28 (14.1)	17 (12.4)	10 (17.9)	23 (9.9)	16 (9.5)	7 (10.9)
No HR functions	23 (11.6)	15 (10.9)	7 (12.5)	3 (1.3)	0 (0.0)	3 (4.7)
Other***	5 (2.5)	2 (1.5)	3 (5.3)	7 (3.0)	3 (1.8)	4 (6.3)
Missing	1 (0.5)	0 (0.0)	0 (0.0)	0 (0.0)	0 (0.0)	0 (0.0)
*Current employer: Sector^2^*						
For-profit sector	130 (65.7)	87 (63.5)	41 (73.2)	174 (74.7)	121 (72.0)	52 (81.3)
Non-profit sector	50 (25.2)	37 (27.0)	12 (21.4)	55 (23.6)	45 (26.8)	10 (15.6)
Public sector	9 (4.5)	6 (4.4)	3 (5.4)	4 (1.7)	2 (1.2)	2 (3.1)
Other****	8 (4.0)	7 (5.1)	0 (0.0)	0 (0.0)	0 (0.0)	0 (0.0)
Missing	1 (0.5)	0 (0.0)	0 (0.0)	0 (0.0)	0 (0.0)	0 (0.0)
*Current employer: Size^2^*						
Micro (10 > employees)	8 (2.7)	5 (3.2)	3 (5.0)	14 (6.0)	5 (3.0)	9 (14.1)
Small (10–99 employees)	22 (7.5)	14 (8.9)	7 (11.7)	67 (28.8)	48 (28.6)	19 (29.7)
Medium (100–999 employees)	69 (23.5)	54 (34.4)	14 (23.3)	87 (37.3)	67 (39.9)	20 (31.3)
Large (1,000–4,999 employees)	51 (17.3)	36 (22.9)	13 (21.7)	16 (6.9)	12 (7.1)	4 (6.3)
Very large (5,000 < employees)	47 (16.0)	28 (17.8)	19 (31.7)	49 (21.0)	36 (21.4)	12 (18.8)
Missing	97 (33.0)	20 (12.7)	4 (6.7)	0 (0.0)	0 (0.0)	0 (0.0)

^1^Includes participants who did not disclose gender

^2^Only asked of participants who were employed full time or part time (*n* = 198 in Study 1, *n* = 233 in Study 2)

*"Other" employment status, e.g., temporary employment

**"Other" role, e.g., "business owner"

***"Other" HR role rank, e.g., "manager"

****"Other" sector, e.g., political campaign.

After providing written informed consent online, participants read a vignette (described in full in S1 Appendix in S1 File) in which a hiring manager was in charge of recruiting a new Vice President (VP) of Operations for a technology company, adapted from Vial et al. [12]. This kind of context might be especially relevant to HR specialists seeking to curb employment discrimination of women, given its focus on an industry (technology) and a high-status position (a VP) in which women are greatly underrepresented [38]. For all participants, the new hire would report directly to a male Chief Executive Officer (CEO), the “third party,” who was described as holding beliefs indicative of bias against women [39]. Participants learned that the hiring manager had found two highly qualified candidates, “Brian N.” and “Karen R.,” who were comparable in terms of personality traits, aptitudes, education, and professional experience in the technology industry. Then, participants learned that, “Given the CEO’s conservative views, the hiring manager decided to select Brian N. as new VP of Operations.” After this, participants completed the attention checks and measures described below.

In addition to these questions, we measured in both studies participants’ tendency to respond in socially desirable ways to address a potential concern that people will fake their responses to appear less biased [40]. We used a 10-item version of the Marlowe-Crowne Social Desirability Scale [41], originally validated by Strahan and Gerbasi [42]. Whereas the 10 items had good reliability in recent investigations (e.g., *Cronbach’s* α = .80 in Grougiou et al., [43]; and α = .65-.75 in Kapoutsis et al., [44]), reliability in our samples was low (α = .57, in Study 1, and α = .58, in Study 2). Thus, we will not discuss this measure further—however, we report ancillary analyses for Study 1 adjusting for social desirability scores in S1 Supplement in S1 File.

For all measures described below, items were rated from 1 (*strongly disagree*) to 5 (*strongly agree*), unless otherwise stated.

#### Attention checks

We asked two questions to identify inattentive participants: (a) “Who did the hiring manager decide to select for VP of operations?” (Brian N. / Karen R.) and (b) “Why did the hiring manager decide to select this candidate?” (Because of the candidate’s qualifications / Because of the views of the CEO.)

#### Perceived acceptability of bias accommodation

We assessed participants’ perceived acceptability of bias accommodation with eight items presented in random order. Four of these items focused on the target’s pragmatic motivations [45], e.g., “The hiring manager acted based on what was reasonable.” We supplemented these items with another four items tailored to the current investigation: (a) The hiring manager made the right decision; (b) The hiring manager’s decision was justified; (c) The decision that the hiring manager made was acceptable; and (d) It was wrong for the hiring manager to make this particular decision (reverse-scored). We conducted an exploratory factor analysis (EFA) with principal axis factor (PAF) extraction to examine the underlying structure of the perceived acceptability construct, employing a Promax rotation to allow factors to correlate with one another [46]. After removing the reverse-scored item, which had low communality < .20 [47], results revealed a single factor with eigenvalue > 1 [48, 49], which was confirmed by a scree test [50], and explained 54.81% of variance (loadings .64-.82). We averaged the seven items into a measure of perceived acceptability (α = .89).

#### Perceived normativity of bias accommodation

Participants rated three statements assessing the perceived prevalence of third-party bias accommodation in hiring selections, e.g., “Most hiring managers would act in a similar way as the hiring manager in the story”. An EFA with PAF extraction and Promax rotation on the three items revealed a single factor that explained 58.64% of variance (loadings .61-.88) based on eigenvalues and the scree plot [48–50]. We averaged the three items into a single measure of perceived normativity of bias accommodation, α = .80.

#### Role demand to prioritize candidate fit with others

Participants completed four different measures reflecting the role demand for hiring managers to prioritize fit with others when selecting job candidates on a scale ranging from 1 (*strongly disagree*) to 7 (*strongly agree*). One measure included three items representing a general role expectation to consider the preferences of existing organizational members when making hiring decisions (e.g., “When evaluating job candidates for a new position, anticipating the preferences of existing company members should be a top priority for recruiters / hiring managers”). Participants also rated (1–5) nine items adapted from Cable and DeRue [51], which specified role responsibilities to maximize fit between a new candidate and (a) the supervisor, three items; (b) potential co-workers, three items; and (c) the organization as a whole, three items. A sample item is, “The recruiter/hiring manager is responsible for finding candidates whose values are very similar to the values of [the supervisor/coworkers/the organization].” All twelve items on role demand endorsement were presented together in random order. For half of the sample, these items were presented before the vignette, whereas the other half of the sample these questions appeared after the vignette.

We submitted the twelve items to an EFA with PAF extraction and Promax rotation, which revealed two factors based on eigenvalues and examination of the scree plot [48–50]. The first factor contained the nine items on general fit with others, fit with supervisor, and fit with co-workers (44.24% of variance; loadings .64-.80), which we standardized and averaged into a *general role demand* composite (α = .91). The second factor included the three items on *person-organization fit* (11.97% of variance; loadings .74-.83), which were averaged together (α = .80).

#### Role-related concerns

Participants were asked to imagine that the hiring manager in the narrative selected the female candidate despite knowing about the CEO’s gender views (i.e., did not accommodate bias), and to rate three sets of statements denoting (a) interpersonal concerns, (b) task-focused concerns, and (c) professional concerns, in random order.

To measure *interpersonal concerns*, we used three items from Vial et al. [12] referring to potential interpersonal issues if the female candidate were selected, e.g., “The CEO would not respect Karen R. as VP of Operations” (α = .75). To measure *task-focused concerns*, we used three items from Vial et al. [12] referring to potential task-related issues if the female candidate were selected, e.g., “Karen R. would have difficulties performing at a high level” (α = .74). To measure *professional concerns*, we developed four items that measured concerns about the hiring managers’ professional standing were he to select the female candidate, e.g., “The hiring manager’s credibility in the company might suffer” (α = .72). An EFA with PAF extraction and Promax rotation on the ten items revealed three factors with eigenvalues > 1 [48, 49], which were confirmed by a scree test [50]. The three factors correspond to interpersonal concerns (26.83% of variance; loadings .53-.88), task-focused concerns (12.95% of variance; loadings .64-.83), and professional concerns (9.05% of variance; loadings .48-.82). Items within each factor were averaged into three separate measures.

#### Social Dominance Orientation (SDO)

To test the hypothesis H4 that egalitarian ideals are negatively correlated with finding it acceptable for someone to accommodate third-party bias, we used an 8-item scale (α = .76) validated by Ho et al. [52], rated from 1 (*strongly oppose*) to 7 (*strongly favor*), to measure Social Dominance Orientation (SDO) [53], an individual difference variable that predicts discriminatory hiring decisions [37] and tolerance of bias expressions [54]. We conducted an EFA with PAF extraction and Promax rotation on the eight items. After removing three items with communalities < .20 [47], results revealed a single factor with eigenvalue > 1 [48, 49], which was confirmed by a scree test [50], and explained 45.10% of variance (loadings .58-.74). We averaged the five items into a measure of social dominance orientation (α = .78).

#### Professional experience with third-party bias influence

We asked participants to indicate how common it is, in their own experience, for clients to give explicit instructions to professionals in charge of recruiting staff not to hire someone from a certain group, from 0 (*less common*, *0/10 clients*) to 10 (*more common*, *10/10 clients*). We specified that “client” meant “the person or persons who give the professional the task of recruiting, selecting, and hiring someone to fill a given vacancy.” We then asked participants to indicate how common it is for professionals in charge of recruiting staff to infer or assume, in the absence of explicit instructions, that a client would not want to hire someone from a certain social group, from 0 (*less common*, *0/10 clients*) to 10 (*more common*, *10/10 clients*).

The study concluded with demographic questions (e.g., age, gender). Prior to debriefing, we asked participants, with assurance that they would still be allowed to enter the voucher lottery, to indicate whether any of their answers were random or meant as jokes (yes/no). Those who answered “yes” (*n* = 11) were excluded from analysis.

### Results

We conducted a confirmatory factor analysis (CFA) with maximum likelihood estimation using the *sem* command in StataSE (version 16) to examine the construct structure underlying the scenario-based measures (i.e., excluding social dominance orientation). We employed a Satorra–Bentler adjustment to obtain estimates that are robust to nonnormality [55, 56; see also 57] due to some concerns with skewness in our data (see S1 Supplement in S1 File). This CFA provided support for the 7-factor model, indicating the distinctiveness of the seven constructs: (a) perceived acceptability, (b) perceived normativity, (c) general role demand composite, (d) person-organization fit, and (e) interpersonal, (f) task-focused, and (g) professional role-related concerns. The scaled chi-square value for the 7-factor model, *χ*^2^ = 664.35, *df* = 443, *p* < .001, was lower than that for a saturated model, *χ*^2^ = 3583.24, *df* = 496, *p* < .001, and fit indices indicated that a 7-factor model fit the data well (TLI = .920, CFI = .928, RMSEA = .047, SRMR = .054).

As seen in Table 2, which presents bivariate correlations between all variables in Study 1, the measures of perceived acceptability and normativity of bias accommodation converged but were not redundant at *r* = .35. Similarly, the two measures of role demand endorsement (general role demand composite and person-organization fit), were positively correlated at *r* = .31, indicating convergence without redundancy. Finally, the same was the case for the three role-related concerns measures (correlations from *r* = .19 to *r* = .38). These role-related concerns measures, which capture context-specific concerns about the role, diverged from the general role demand composite and person-organization fit measure (which capture a more stable role orientation), *r* = .01 to *r* = .12, demonstrating the discriminant validity of these measures.

**Table 2:** Bivariate correlations among variables in Study 1.

	01	02	03	04	05	06	07	08	09	10	11	12	13
01. Perceived acceptability	--												
02. Perceived normativity	.36***	--											
03. General role demand composite	.25***	.14*	--										
04. Person-organization fit	.02	.05	.31***	--									
05. Interpersonal concerns	.19**	.32***	.12†	.05	--								
06. Task-focused concerns	.24***	.22***	.03	-.02	.19**	--							
07. Professional concerns	.18**	.32***	.05	.01	.38***	.20**	--						
08. Prevalence of explicit client requests	-.03	.29***	.09	.03	.17*	-.03	.20**	--					
09. Prevalence of inferred client preferences	.05	.43***	.11	.05	.16*	-.02	.22**	.58***	--				
10. Years of HR experience	-.11	-.15*	-.08	-.07	-.16*	.10	-.14*	-0.1	-.17*	--			
11. Positions filled last year	-.04	.09	.04	-.04	.12	.05	-.02	.12†	.09	-.05	--		
12. Participant age	-.03	-.09	-.16*	-.06	-.04	.12†	-.13†	-.15*	-.05	.55***	-.25**	--	
13. Participant gender (0 = M, 1 = F)	-.15	-.004	-.04	-.04	.12†	-.12†	.05	.05	.002	.03	.01	-.12†	--
14. Social dominance orientation	.20**	-.05	.04	-.07	-.09	.09	.07	.04	-.04	.07	-.04	-.10	-.05

^†^*p* < .10

*p < .05

** *p* ≤ .01

*** *p* ≤ .001

#### Association between role demand endorsement and the perceived acceptability and normativity of bias accommodation

As reported in Table 2, for the composite measure of general endorsement of the role demand to maximize candidate fit with others, results revealed significant, positive correlations with (a) acceptability, *p* < .001 (*d* = .52), and (b) normativity, *p* = .036 (*d* = .28), consistent with H1a and H1b. For the measure of endorsement of the role demand to maximize candidate fit with the organization, these correlations were not significant (acceptability, *p* = .736, *d* = .04; normativity, *p* = .464, *d* = .10). Thus, we did not examine this measure further.

#### Association between role-related concerns and perceived acceptability and normativity of bias accommodation

Consistent with H2a and H2b, role-related concerns were positively associated with ratings of (a) acceptability and (b) normativity, as reported in Table 2: Interpersonal concerns (acceptable, *p* = .003, *d* = .39; normative, *p* < .001, *d* = .68); task-focused concerns (acceptable, *p* < .001, *d* = .49; normative, *p* = .001, *d* = .45); and professional concerns (acceptable, *p* = .006, *d* = .37; normative, *p* < .001, *d* = .68).

#### Perceived acceptability of bias accommodation as a function of individual difference factors and role demand endorsement

**Fig 1:**
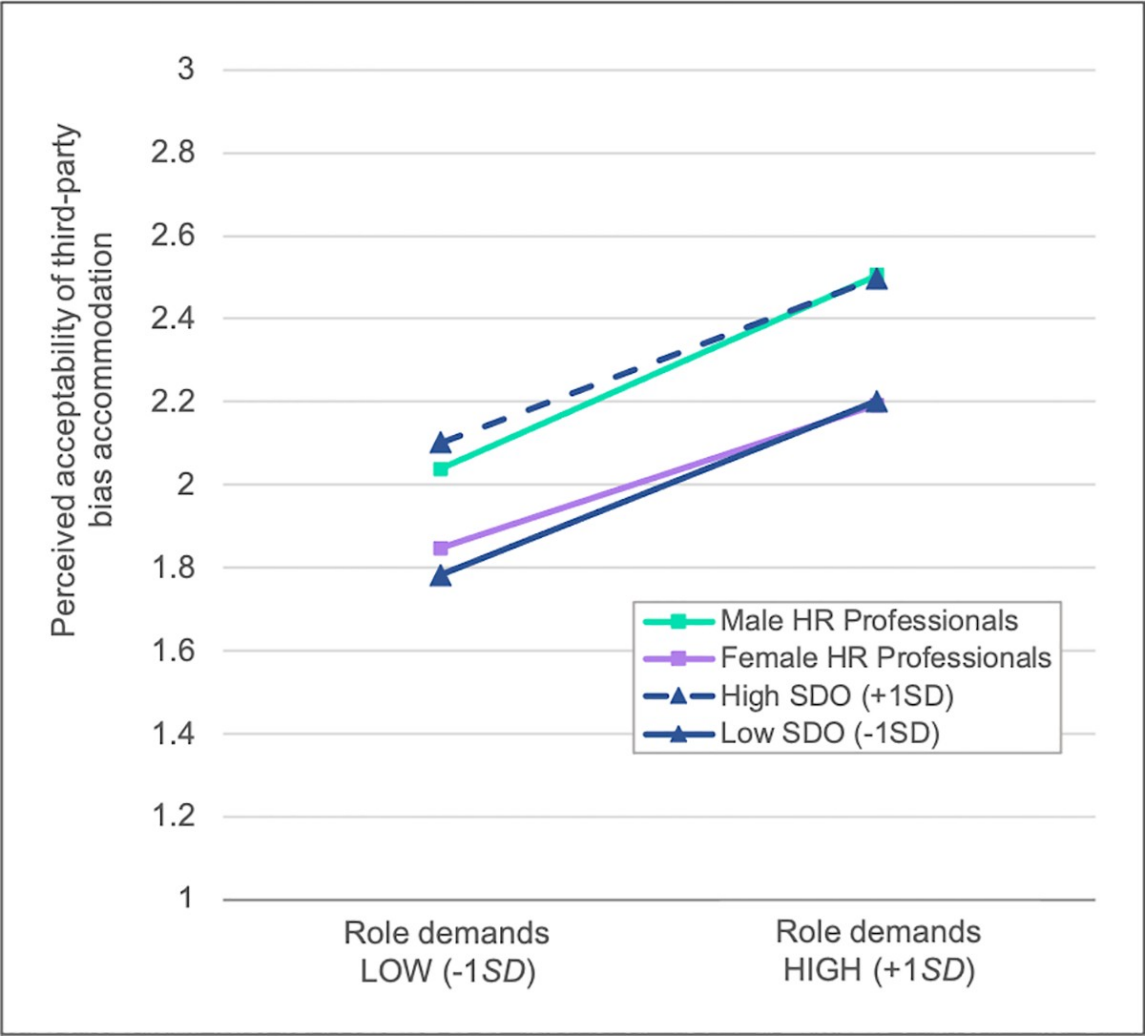
Perceived acceptability of bias accommodation from a vicarious perspective in Study 1. Responses are plotted as a function of endorsement of the role demand to consider candidate fit with others (general role demand composite), at relatively low and high levels (i.e., +/-1 *SD* above/below mean), separately for male (green line) and female (purple line) HR professionals, and for HR professionals with relatively low (dashed blue line) and relatively high (solid blue line) social dominance orientation (SDO) scores (i.e., +/-1 *SD* above/below mean).

Given that perceived acceptability correlated significantly with the general role demand composite—but not with person-organization fit, we focused on the former to test H3 and H4. Specifically, we regressed acceptability of bias accommodation on participant gender (-1 = male, 1 = female), SDO scores (mean-centered), the general role demand composite (mean-centered), and the two-way interactions between general role demand and participant gender and between general role demand and SDO. Results, plotted in Fig 1, revealed the expected significant coefficient of general role demand endorsement, *b* = .26, *SE* = .08, *p* = .001, β = .24: Stronger endorsement was associated with finding the decision more acceptable (supporting H1a). The participant gender coefficient was also significant, *b* = -.13, *SE* = .06, *p* = .043, β = -.13: Female participants overall found it less acceptable than male participants when another hiring manager accommodated bias against women, in line with H3. Similarly, in line with H4, the SDO coefficient was significant, *b* = .14, *SE* = .05, *p* = .007, β = .18: Higher SDO was associated with finding bias accommodation more acceptable. There were no significant two-way interactions between general role demand endorsement and individual-level predictors, *p*s > .61.

#### Professional experience with third-party bias influence

Participants estimated that roughly 30% of clients (*M* = 2.96, *SD* = 2.80) make explicit requests not to hire from certain groups (where 0 = *0% of clients* and 10 = *100% of clients*), and that hiring professionals make spontaneous inferences about client biases roughly 30% of the time (*M* = 3.02, *SD* = 2.61). As can be seen in Table 2, perceived prevalence of explicit client requests and of inferences about clients’ biases were significantly, positively correlated; moreover, a repeated-measures Analysis of Variance (ANOVA) comparing the two estimates revealed no significant difference, *F*(1, 231) = .41, *p* = .52, η_*p*_^2^ < .01. Both variables were positively and significantly associated with the perceived normativity of bias accommodation, *p*s <. 001 (*d* = .60, for client requests, and *d* = .95, for spontaneous inferences).

Unexpectedly, professional experience with explicit client requests was negatively associated with age (see Table 2), *p* = .028, *d* = .31. In an effort to better understand HR professionals’ experiences with third-party bias, we conducted additional analyses to examine whether this relationship with age might be partly due to older professionals occupying higher ranks and being less directly involved in hiring relative to younger professionals, which might limit older professionals’ exposure to clients (and their biases). Age and HR rank were positively associated, *r*(183) = .23, *p* = .001, and both age and HR rank were negatively related to the number of positions filled in the previous year, *r*(169) = -.25, *p* = .001, for age, and *r*(160) = -.20, *p* = .012, for HR rank. The correlation between age and explicit client requests was no longer significant when controlling for HR rank and number of positions filled during the previous year, *r*(151) = -.14, *p* = .08.

### Discussion

To our knowledge, these results provide the first line of evidence that certain ways of construing the hiring manager role may lead hiring professionals to condone bias accommodation as more or less required of any role occupant [22, 23]. Specifically, consistent with our hypotheses, participants in Study 1 were more tolerant of bias accommodation to the extent that they endorsed a role priority for hiring managers to maximize candidate fit with others. Indeed, participants found it more acceptable and commonplace for an individual to pass on the biases of a relevant third party to the extent that they viewed it as a primary role responsibility for hiring managers to take into consideration the preferences of others (including the future supervisor and co-workers). Further supporting the centrality of role-related considerations, we also found that role-related concerns (interpersonal, task-focused, and professional) were linked with hiring professionals’ tendency to find bias accommodation by others as acceptable and normative.

To account for individual differences, in Study 1 we examined responses as a function of respondent gender and egalitarian beliefs (SDO). Both of these factors were associated with the acceptability of bias accommodation: Female (vs. male) participants and those with lower SDO (vs. higher SDO), indicative of more egalitarian views, judged it to be less acceptable for a hiring manager to discriminate against a female job candidate due to third-party bias against women. These results align with past findings based on nonprofessional samples, in which women and more egalitarian individuals reported stronger feelings of remorse, guilt, and shame after having accommodated third-party gender bias [12]. However, above and beyond any variance explained by participant gender and SDO scores, endorsement of the role demand to maximize candidate fit with others was independently, positively associated with tolerance of bias accommodation.

Study 1 is the first, to our knowledge, to document hiring professionals’ own experience with third-party bias influence, and the results suggest that they frequently encounter third-party pressures to avoid hiring candidates from specific groups. Insofar as a perception of third-party bias may systematically restrict opportunities for women relative to men [8, 12, 13], these results highlight the crucial part that professionals in roles involving hiring decisions can play in ensuring equitable treatment of women in the workplace. Next, in Study 2, we examine whether hiring professionals are actually vulnerable to accommodating others’ biases when making hiring decisions—and whether such vulnerability might depend on the way that they construe their assigned role.

## Study 2: Do hiring professionals accommodate bias?

In Study 2 we seek insight into whether professionals who are highly knowledgeable of hiring practices are personally vulnerable to accommodating the perceived biases of relevant third parties when making hiring decisions, and whether variability in role construal moderates this effect. We test Hypotheses 1c, 2c, and 2d by asking participants to imagine—this time from the actor’s vantage point—that they were in charge of making a hiring decision involving male and female job candidates. We compared participants’ hiring decisions in a condition in which there were cues to suggest that a relevant third party was biased against women (i.e., in which the CEO is known for his position that women should put family ahead of career) versus a condition in which such bias cues were absent (see Vial et al. [12, 13]). Participants also completed a series of measures, including endorsement of a role demand for hiring managers to prioritize candidate fit with others.

As in Study 1, we examine HR professionals’ own personal experience with third-party requests to discriminate based on demographic characteristics as well as the prevalence of inferred third-party biases. Further extending this inquiry, in Study 2, we also asked participants to indicate which social groups were the targets of explicit demands and inferences. This list included, along with gender groups, other groups (e.g., based on race/ethnicity) that might similarly be the targets of third-party bias and accommodation processes.

### Materials and methods

Participants in the U.S. completed the study online in exchange for a 1/10 chance of winning a $20 gift card. Based on a-priori power analysis (see S2 Supplement in S1 File), we recruited 293 participants through professional networks’ listservs, targeted email invitation campaigns, and chain referrals. Recruitment source did not impact results. We excluded five participants (1.7%) for indicating that their answers were jokes or random, and another four (1.4%) for inattention. The final sample was *n* = 284 (mean age = 44.74, *SD* = 12.05; 72.0% female; 80.0% White). A sensitivity power analysis indicated that this sample size was sufficient to detect small to medium effect sizes (see S2 Supplement in S1 File). Gender was missing for 38 participants; but we included them in any analysis for which gender was not a factor. On average, participants had 14.64 years of experience in HR (*SD* = 9.12). Most (89.5%) were involved in hiring efforts at the time of the study, and over the past year (96.1%), in which they filled or helped fill 26 positions on average (*SD* = 18.66). Detailed sample characteristics appear in Table 1.

After providing written informed consent online, participants read a short vignette (similar to the one in Study 1, but from an actor’s perspective; see S2 Appendix S1 File) which asked participants to imagine that they worked at a recruitment agency that had been hired by a technology company to assist in recruiting a Vice President (VP) of Operations. The VP would report to a male Chief Executive Officer (CEO) (who was the “third party”), and work closely with him. We manipulated cues to third-party bias against women by varying information about the background and gender-related beliefs of the third party [39; see also 12, 13]. Half of participants were randomly assigned to a condition in which the CEO was described as holding bias against career women (e.g., “He believes that it is important for women to put families before careers”). For the other half of participants, the CEO’s description provided details about his education and background only.

After the vignette, participants saw the profiles of three candidates for the position (e.g., degree, previous job). There were two strong candidates, a man and a woman (the targets of interest), and a third candidate who was comparatively under-qualified (i.e., a foil), which we counterbalanced to be either male or female. We included this foil to disguise the relevance of candidate gender, enhance the realism of the task, and identify inattentive participants (which were excluded from analysis). Very few participants (1.4%) selected the foil, suggesting that, as intended, the foil was perceived as being comparatively less qualified than the other two candidates. We conveyed candidate gender with first names (“Karen R.” and “Brian N.” for the strong candidates; “Ann S.” or “John S.” for the foil), as in prior research [58, 59]. The profiles also included a box ostensibly reserved for a photo showing a male/female silhouette with the notice “*Image not available*.” The three candidate profiles appeared on the screen simultaneously, and we counterbalanced their order from left to right, as well as which of the two strong profiles belonged to a male or a female candidate.

#### Candidate preferences

For each of the three possible candidate pairs (i.e., Karen-Brian, Karen-foil, Brian-foil), participants were asked their hiring preference, with the ratings for the two strongly and equivalently qualified female and male candidates (Karen and Brian) of direct relevance to our hypotheses (e.g., “Between Brian N. and Karen R., who would you select?”). We did not analyze the comparisons involving the foil. Participants indicated their preference on a scale from 1 (*definitely select Brian N*.) to 9 (*definitely select Karen R*.). These preference ratings were the central outcome in Study 2, along with participants’ final candidate selection.

#### Final candidate selection

Participants were asked to make a final decision to select one out of the three candidates (i.e., a zero-sum choice). We marked participants who selected the foil (1.4%) as “inattentive” and removed them from analysis.

#### Role-related concerns

Participants indicated how much they agreed or disagreed (from 1 to 5) with six items [12] reflecting role-related concerns about hiring a female candidate. Three items measured interpersonal concerns (α = .76). For task-focused concerns, we included three items; however, we removed one item (“In this situation, a woman would be a successful VP of Operations” [reverse-scored]) to improve scale reliability from α = .66 to α = .73. The two sets of items, as well as the items within each set, were randomized.

#### Bias cues manipulation check

Participants rated the following statement from 1 (*strongly disagree*) to 6 (*strongly agree*): “*The CEO is biased against working women*”.

#### Role demand to prioritize candidate fit with others

We used the first, three-item measure of role demand endorsement that we employed in Study 1 (α = .90).

#### Professional experience with third-party bias influence

We asked participants how common it is for clients to request that HR professionals in charge of recruiting staff not hire someone from a certain group, from 0 (*less common*, *0/10 clients*) to 10 (*more common*, *10/10 clients*) (*n* = 262; range: 0–10; *M* = 2.79, *SD* = 2.59). We then asked participants when was the last time that they received such a direct request, and which candidate group(s) they were instructed to avoid hiring. Then, we asked participants the same questions, modified to refer to instances in which HR professionals would assume that a client would not want to hire from a certain group in the absence of explicit client requests, from 0 (*less common*, *0/10 clients*) to 10 (*more common*, *10/10 clients*) (*n* = 258; range: 0–10; *M* = 3.14, *SD* = 2.65).

As in Study 1, in addition to these questions, we measured participants’ tendency to respond in socially desirable ways [40, 41]. However, we excluded this measure from analysis due to low reliability (α = .57, in Study 1, and α = .58, in Study 2). Ancillary analyses for Study 2 adjusting for social desirability scores are reported in S2 Supplement in S1 File, for interested readers. The study concluded with demographic questions (e.g., age, gender). Prior to debriefing, we asked participants, with assurance that they would still be allowed to enter the gift card lottery, to indicate whether any of their answers were random or meant as jokes (yes/no). Those who answered “yes” (*n* = 5) were excluded from analysis.

### Results

We first examine participants’ own professional experience with third-party bias influence, drawing comparisons with the responses of Irish participants in Study 1. Then, we dive into the results of the experiment.

#### Professional experience with third-party bias influence

Preliminary analysis indicated that responses to the professional experience questions were unaffected by experimental condition (see S2 Supplement in S1 File). As in Study 1, results in Study 2 suggest that HR professionals might frequently encounter third-party pressures to avoid hiring candidates from specific groups. Participants estimated that close to 28% of clients (*M* = 2.79, *SD* = 2.59) make explicit requests to avoid hiring from certain groups (where 0 = *0% of clients* and 10 = *100% of clients*), and that hiring professionals make spontaneous inferences about client biases more than 30% of the time (*M* = 3.14, *SD* = 2.65). Unlike participants in Study 1, participants in Study 2 estimated that spontaneous inferences were significantly more common than explicit client requests, *F*(1, 256) = 5.99, *p* = .015, η_*p*_^2^ = .023 (*d* = .31). However, similar to participants in Study 1, the two estimates were significantly correlated, as can be seen in Table 3, which presents bivariate correlations between all variables in Study 2.

**Table 3:** Bivariate correlations among variables in Study 2.

	01	02	03	04	05	06	07	08	09	10	11
01. Bias cues condition (0 = control, 1 = third-party bias)	--										
02. Participant gender (0 = M, 1 = F)	--	--									
03. Candidate preference (higher favor female)	-.04	.06	--								
04. Final candidate selection (0 = M, 1 = F)	-.12†	.01	.70***	--							
05. Role demand to prioritize fit with others	.01	-.05	-.16*	-.16*	--						
06. Interpersonal concerns	.47***	.09	-.13*	-.20**	-.03	--					
07. Task-focused concerns	.18**	-.03	-.09	-.16**	.04	.36***	--				
08. Prevalence of explicit client requests	.09	.03	-.07	-.09	.11†	.08	.03	--			
09. Prevalence of inferred client preferences	.11†	.04	-.01	-.03	.06	.11†	.06	.49***	--		
10. Years of HR experience	.08	.04	.09	.05	-.13*	.03	.10	.01	-.08	--	
11. Positions filled last year	.10	.01	-.04	-.10	-.11	.01	-.04	.02	.02	-.07	--
12. Participant age	.01	-.08	.01	.04	-.11†	-.04	.05	-.07	-.17**	.67***	-.13†

^†^*p* < .10

*p < .05

** *p* ≤ .01

*** *p* ≤ .001

Extending the scope of our inquiry beyond Study 1, we also asked participants how often they experience the influence of third-party biases in their own professional lives. Results reveal that up to 46% of participants reported receiving explicit requests from their own clients, with most of those requests (47.1%) taking place within the previous year, 24.8% between 1 and 4 years ago, and the rest (28.1%) 5 or more years ago. Moreover, 51.5% of respondents reported having personally inferred client preferences in the absence of direct requests, mostly during the previous year (41.3%), and 29.3% each between 1–4 years ago and 5 or more years ago. Among participants who reported never having received explicit client requests to discriminate, 36.2% report having personally inferred client biases. Similar to Study 1, professional experience with inferring clients’ biases was negatively associated with age (as seen in Table 3), *p* = .009, *d* = .34. Age and HR rank were positively associated, *r*(225) = .34, *p* < .001, and both were negatively related to the number of positions filled in the previous year, *r*(216) = -.13, *p* = .055, for age, and *r*(215) = -.28, *p* < .001, for HR rank. However, he correlation between age and inferences of client biases remained significant when controlling for HR rank and number of positions filled during the previous year, *r*(204) = -.20, *p* = .003.

Gender-based groups were the most common targets for both explicit client instructions (60.3%) and spontaneous inferences of client preferences (64.7%), as reported in S1 Table in S1 File. The next most common social category was race or ethnicity-based (33–41.3%), followed by age-based (19.8–11.3%), and religion-based (5.8–10.5%). One participant reported receiving explicit requests to avoid hiring gay/lesbian candidates. Of note, with the exception of age-based discrimination, spontaneous inferences were more common than explicit requests for all social category groups. This discrepancy was particularly large for transgender candidates, candidates from certain religions, sexual minority candidates, and Arab candidates.

While primarily descriptive, these results constitute the first evidence that phenomena like the “third-party prejudice effect” [12, 13] and anticipatory sorting [8], which have been studied solely, to our knowledge, in the context of gender-based employment discrimination, may be highly relevant to understanding employment discrimination based on other group-based factors besides gender—a possibility worthy of consideration in future investigations.

#### Bias cues manipulation check

A 2 (bias cues condition: third-party bias vs. no cues) *×* 2 (participant gender: male vs. female) ANOVA revealed a significant effect of condition on participants’ perceptions that the third party was biased against working women, *F*(1, 242) = 169.34, *p* < .001, η_*p*_^2^ = .41 (*d* = 1.67). As intended, participants rated the third party as more biased in the third-party bias cues condition (*M* = 4.40, *SD* = 1.36) versus the no cues condition (*M* = 2.05, *SD* = 1.08). There was also a significant effect of participant gender, *F*(1, 242) = 6.47, *p* = .012, η_*p*_^2^ = .03 (*d* = .33): Female participants perceived the third party as more biased (*M* = 3.31, *SD* = 1.68) than male participants (*M* = 3.04, *SD* = 1.74). The interaction was not significant, *F*(1, 242) = 2.74, *p* = .10, η_*p*_^2^ = .01.

#### Candidate preferences

We first corroborated that bias cues condition (third-party bias vs. no cues) had no significant impact on the moderator, endorsement of the role demand to prioritize candidate fit with others, *F*(1, 242) = .13, *p* = .72, η_*p*_^2^ < .01. Thus, we examined whether endorsement of this role demand moderated the third-party bias effect.

To do this, we regressed preference for a female candidate over a male candidate on experimental condition (-1 = no cues, 1 = third-party bias), participant gender (-1 = male, 1 = female), role demand endorsement (mean-centered), and all interactions. In contrast with previous work with lay samples [12, 13], in this study with HR professionals the main effect of experimental condition was not significant, *b* = -.06, *SE* = .16, *p* = .72, β = -.03. However, as expected, there was a significant two-way condition × role demand endorsement interaction, *b* = -.25, *SE* = .10, *p* = .009, β = -.19: Consistent with H1c, when third-party bias cues were present, participants who reported stronger endorsement of the role demand to maximize candidate fit with others had significantly lower preference for a female candidate, *b* = -.47, *SE* = .14, *p* = .001, β = -.35. When third-party bias cues were absent, role demand endorsement was unrelated to candidate preferences, *b* = .04, *SE* = .13, *p* = .75, β = .03.

**Fig 2:**
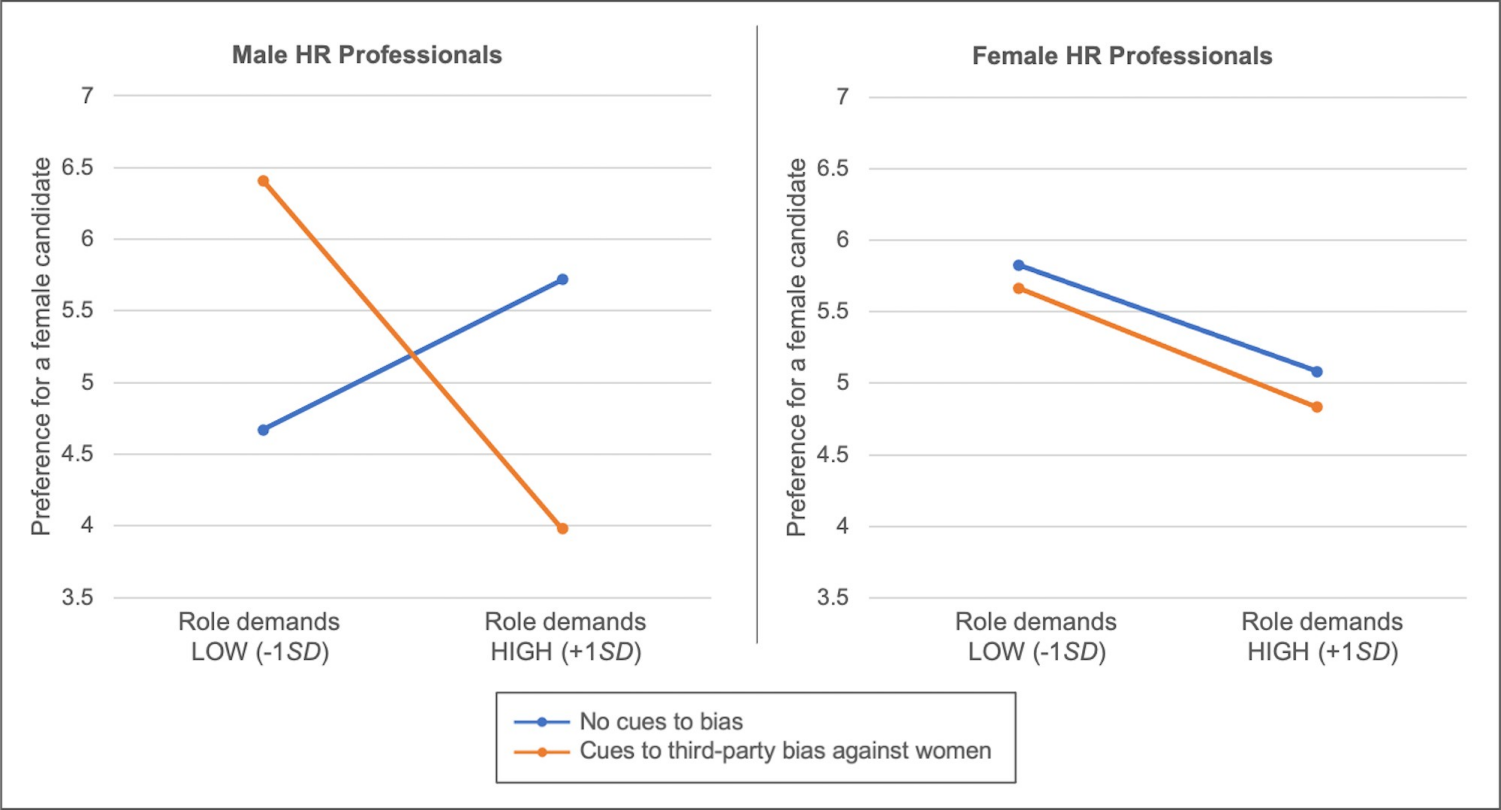
Preference for a female candidate over a male candidate in Study 2. Responses are plotted as a function of bias cues condition (third-party bias against women, orange line; no bias, blue line) at relatively low and high levels (i.e., +/-1 *SD* above/below mean) of endorsement of the role demand to consider candidate fit with others (general role demand composite), separately for male HR professionals (left panel) and female HR professionals (right panel).

This two-way interaction was qualified by a significant three-way, condition *×* role demand endorsement *×* participant gender interaction, *b* = .24, *SE* = .10, *p* = .012, β = .18. To decompose this three-way interaction (plotted in Fig 2), we examined the two-way interaction between role demand endorsement and experimental condition separately for male and female participants. For male participants, there was a significant two-way condition *×* role demand endorsement interaction, *b* = -.99, *SE* = .33, *p* = .003, β = -.51: In the third-party bias condition, male participants with stronger role demand endorsement had significantly lower preference for the female candidate, *b* = -.70, *SE* = .24, *p* = .004, β = -.52. In the control condition, male participants with stronger role demand endorsement had a slightly higher (nonsignificant) preference for a female candidate, *b* = .30, *SE* = .23, *p* = .19, β = .22. For female participants, there was no significant two-way condition × role demand endorsement interaction, *b* = -.02, *SE* = .20, *p* = .90, β = -.01; no significant effect of role demand endorsement, *b* = -.21, *SE* = .13, *p* = .12, β = -.16; and no significant effect of condition, *b* = -.11, *SE* = .17, *p* = .51, β = -.05. Thus, for the continuous measure of preference for a female over a male candidate, results lend support to H1c but only among male participants: Their responses to the perceived gender biases of relevant organizational members were moderated by the extent to which they endorsed the hiring manager’s role priority to maximize candidate fit with others. Next, we examined participants’ final candidate selections as a binary choice.

#### Final candidate selection

**Fig 3:**
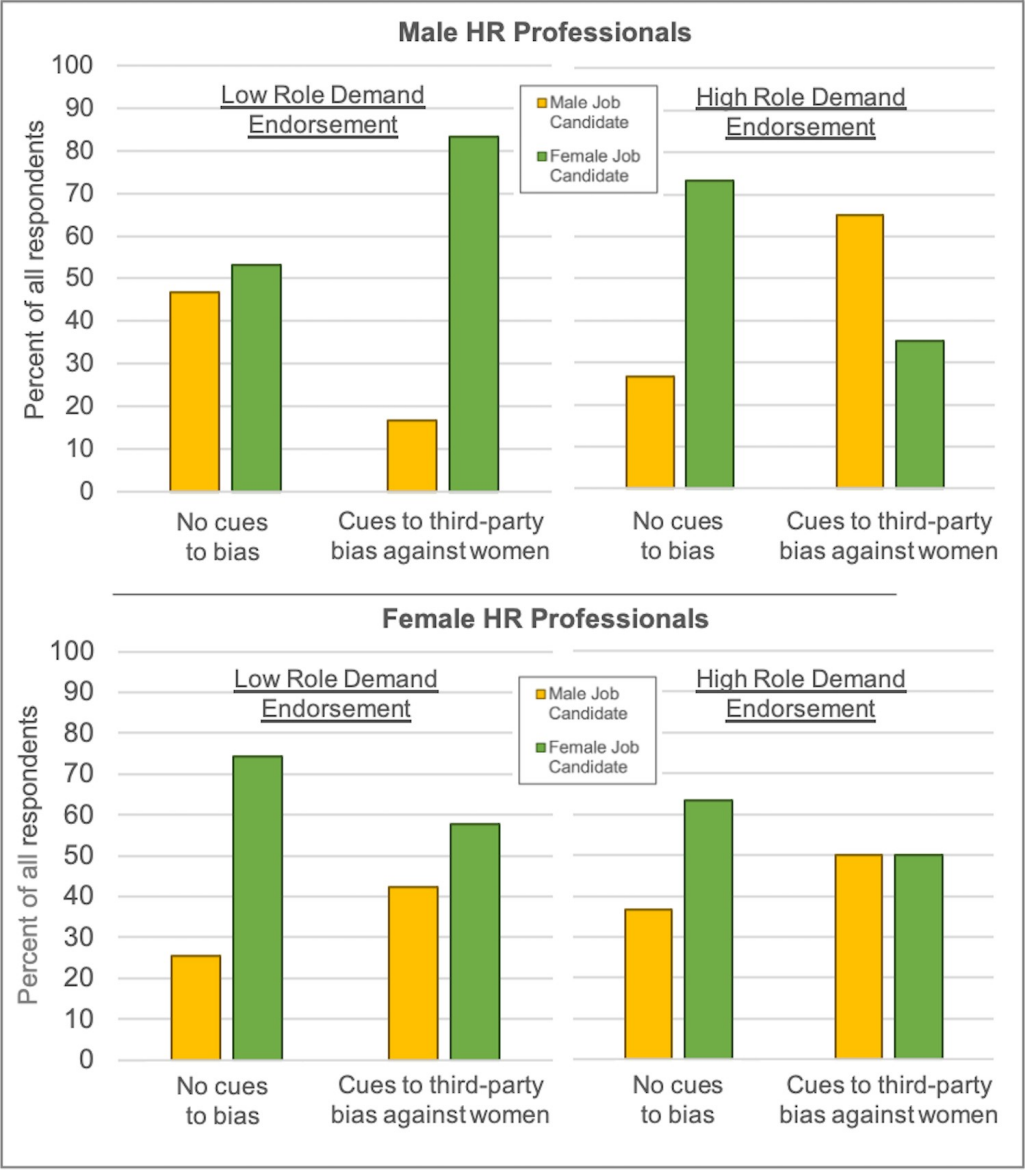
Final selection of male and female job candidates in Study 2. Percent of participants who selected the male candidate (yellow bars) and the female candidate (green bars) as a function of bias cues condition (x-axis) at relatively low (left side) and high (right side) levels of endorsement of the role demand to consider candidate fit with others (general role demand composite), plotted separately for male HR professionals (top panel) and female HR professionals (bottom panel).

We conducted a binary logistic regression on participants’ final selection of a female candidate (coded as 1) or a male candidate (coded as 0). As in the previous analysis, we included experimental condition (-1 = no cues, 1 = third-party bias), participant gender (-1 = male, 1 = female), role demand endorsement (mean-centered), and all interactions as predictors. Again, this model revealed no significant main effect of experimental condition, *b* = -.10, *SE* = .17, *p* = .54, similar to the continuous measure of candidate preference. However, consistent with H1c, there was a significant two-way condition × role demand endorsement interaction, *b* = -.32, *SE* = .12, *p* = .006: When third-party bias cues were present, participants with stronger endorsement of the role demand to maximize candidate fit with others were significantly less likely to select the female candidate, *b* = -.65, *SE* = .19, *p* = .001, OR = .52, 95% CI [.36, .77]. When bias cues were absent, role demand endorsement was unrelated to candidate selection, *b* = -.008, *SE* = .12, *p* = .95. These results mirror the pattern of responses on the continuous measure of candidate preference. Moreover, similar to that analysis, the two-way condition × role demand endorsement interaction in the binary logistic regression model was qualified by a significant three-way interaction with participant gender, *b* = .26, *SE* = .11, *p* = .026, OR = 1.29, 95% CI [1.03, 1.62]. To decompose this interaction (plotted in Fig 3), we examined the final candidate selections of male and female participants separately. For male participants, the two-way condition *×* role demand endorsement interaction was significant, *b* = -.58, *SE* = .21, *p* = .006. In line with H1c, male participants in the bias cues condition were significantly less likely to select a female candidate the more they endorsed the role demand to prioritize candidate fit with others, *b* = -1.11, *SE* = .36, *p* = .002, OR = .33, 95% CI [.16, .67]. In the control condition, male participants with stronger role demand endorsement were slightly (but not significantly) more likely to select a female candidate, *b* = .04, *SE* = .21, *p* = .83. For female participants, the two-way condition *×* role demand endorsement interaction was not significant, *b* = -.06, *SE* = .10, *p* = .51. Instead, there was a significant main effect of experimental condition: Female participants were less likely to select a female candidate when there were cues to third-party bias (vs. no cues), *b* = -.33, *SE* = .16, *p* = .038, OR = .72, 95% CI [.52, .98], irrespective of role demand endorsement.

#### Role-related concerns

To test H2c that role-related concerns would be higher in the presence of bias cues, we repeated the same regression model twice, first on (a) interpersonal concerns, and then on (b) task-focused concerns, with condition (-1 = no cues, 1 = third-party bias), participant gender (-1 = male, 1 = female), and their interaction. First, for interpersonal concerns, the model revealed as expected a significant effect of condition: Interpersonal concerns about hiring a female candidate were higher in the third-party bias cues condition (vs. control), *b* = .40, *SE* = .05, *p* < .001, β = .46. There was also a main effect of participant gender: Female (vs. male) HR professionals had stronger interpersonal concerns regardless of experimental condition, *b* = .12, *SE* = .05, *p* = .026, β = .13. The interaction between the two predictors was not significant, *p* = .53. Second, for task-focused concerns, the model similarly revealed a significant effect of condition, *b* = .14, *SE* = .06, *p* = .021, β = .16: Task-focused concerns about hiring a female candidate were higher in the third-party bias cues condition (vs. control). No other effects were significant, *p*s > .53.

To further test H2d that role-related concerns underlie HR professionals’ tendency to accommodate third-party bias, we conducted mediation analyses with 10,000 bootstraps (95% bias corrected) using Model 4 in the SPSS PROCESS macro [60]. To begin, we entered condition as the predictor (0 = control, 1 = third-party bias cues) and participants’ relative preference for a female candidate over a male candidate as the outcome, with the two sets of role-related concerns (interpersonal, task-focused) as parallel mediators and participant gender as covariate. This model revealed mediation by neither interpersonal concerns, *b* = -.29, *SE* = .19, 95% CI[-.66, .08], nor task-focused concerns, *b* = -.04, *SE* = .06, 95% CI[-.18, .07].

We then tested the same mediation model with participants’ final selection of a female candidate (coded as 1) or a male candidate (coded as 0) as the outcome. Unlike the first model on relative hiring preferences, this model predicting final selections showed significant indirect effects of experimental condition on candidate selection via interpersonal concerns, *b* = -.44, *SE* = .20, 95% CI[-.89, -.10], and via task-focused concerns, *b* = -.13, *SE* = .08, 95% CI[-.33, -.02]. We tested whether role demand endorsement might moderate these mediation effects, but we found no evidence of moderated mediation (Model 8 [60]) by interpersonal concerns for relative candidate preferences, *b* = -.01, *SE* = .03, 95% CI[-.052, .062], or for final candidate selections, *b* = -.01, *SE* = .04, 95% CI[-.078, .090]. Nor was there moderated mediation by task-focused concerns for relative candidate preferences, *b* = -.01, *SE* = .01, 95% CI[-.025, .028], or for final candidate selections, *b* = -.01, *SE* = .03, 95% CI[-.061, .062]. Thus, independently from participants’ endorsement of specific role demands around maximizing candidate fit with others, cues to third-party bias against women translated into decisions to accommodate such bias to the extent that these cues raised interpersonal and task-focused concerns for role occupants. Taken together, the two mediation models provide partial support for H2d, depending on response modality (relative candidate preferences versus final zero-sum candidate selections).

### Discussion

Study 2 showed that, as expected, in the presence of cues indicative of third-party bias against women, participants who endorsed more (vs. less) strongly the role demand to prioritize candidate fit with others were also less likely to favor a female candidate over an equally-qualified male candidate. For male participants, this was the case for their responses on both the continuous measure of candidate preference and the final (zero-sum) hiring decision. This result aligns with our main proposition that in-role behaviors—including bias accommodation in roles that involve hiring on behalf of others—depend on the way that role occupants construe the formal demands of the role. For female participants, although relative hiring preferences (as a continuous measure) evinced no gender bias, the third-party bias effect emerged in their final hiring choices. This discrepancy may be due to the zero-sum nature of the latter variable (selecting one candidate out of two qualified ones). People tend to express their own biases more strongly in zero-sum decisions compared to non-zero-sum decisions such as a relative preference between two individuals [61], and perhaps this distinction is also relevant when people accommodate (i.e., express) others’ biases.

As a whole, the results of Study 2 on relative candidate preferences and final selection of a single candidate suggest that HR professionals are less prone to accommodating third-party gender-bias than lay persons [12, 13]. Their responses to third-party bias against women were nuanced, and depended both on individual-level factors (i.e., the HR professional’s gender), role-based factors (i.e., how they construe the demands of the hiring manager role), and the kind of evaluation being made (i.e., a comparative assessment of different candidates versus a zero-sum candidate choice). Mediation analysis further indicated that hiring professionals have similar role-related motives for accommodating bias as nonprofessional individuals [12, 13], although they might be more sensitive to response modality, expressing interpersonal and task-focused concerns about candidate fit with others more strongly in zero-sum candidate choices than in preliminary comparative assessments.

## General discussion

The aim of the current research was to examine whether variability in the way that hiring professionals construe the hiring manager role (e.g., the extent to which they endorse a role demand to prioritize candidate fit with others) would systematically influence the degree to which they support spreading others’ biases when making hiring decisions. We found evidence for this proposition among HR professionals charged with making the decision in the context of third-party bias against women (Study 2). We also found support for our hypothesis among observers of other professionals accommodating the gender biases of a third party (Study 1). Understanding responses to others who accommodate bias as a function of the observer’s construal of the hiring manager role, a currently under-explored issue, has particular implications for hiring decisions that involve multiple decision makers [31, 32]—a procedure that is recommended to reduce bias in hiring [62, 63]. Although previous work on attribution processes suggests that there is often a discrepancy between how people respond when involved in the situation (actors) and when observing others in the situation (observers) [17, 18, 33, 34], our results indicate that an individual’s role construal—specifically in terms of prioritizing candidate fit with others—may have a pervasive influence on hiring decisions, shaping both personal decisions and responses to others’ decisions that systematically disadvantage women.

Whereas past research with nonprofessional samples found a direct main effect for the third-party bias cues condition [12, 13], in Study 2 we did not find this effect among HR professionals despite using the same paradigm and having sufficient statistical power. One interpretation is that hiring professionals, on average, might be less prone to accommodating third-party bias against women than nonprofessional samples. This explanation is consistent with a meta-analysis that found less bias in hiring decisions made by experienced professionals (vs. nonprofessional) samples [64]. It is thus possible that in addition to role construal about prioritizing candidate fit, other role demands may be operating in the profession that bear on professionals’ responses to bias accommodation. For instance, hiring professionals who prioritize fairness and impartiality [65, 66] to a greater degree might exhibit a weaker tendency to accommodate others' biases—a possibility that merits further investigation.

Our findings suggest that, at least when evaluating the decisions of another role occupant from an observer perspective (Study 1), person-level factors such as one’s gender or egalitarian beliefs might operate largely independently from individuals’ role construal. However, future research might further consider factors that may moderate the effects of role construal from the actor’s perspective. Although in the current and previous work on gender bias accommodation [12, 13] participant gender does not appear to directly moderate the effect of third-party bias cues, in Study 2 we found a higher-order, participant gender × bias cues condition × role construal interaction, whereby male participants who strongly prioritized candidate fit with others were especially likely to accommodate another person’s bias and hire a man over a woman. For female respondents, bias accommodation was independent from role construal and emerged only for final (i.e., zero-sum) decisions, which typically elicit higher levels of bias than other outcomes that are less restrictive such as relative preferences [61]. Thus, person-level factors (e.g., respondent gender) might interact with role construal to determine responses to third-party bias in certain conditions (i.e., when making decisions) but not others (i.e., when evaluating others’ decisions), or for certain kinds of decisions (zero-sum) but not others (non-zero-sum). Additional research may help clarify these nuances.

Despite these complexities, we found evidence generally consistent with role-based processes underlying the responses of hiring professionals. Beyond role construal, role-related concerns (interpersonal, task-focused, and professional) were, as expected, associated with finding bias accommodation more acceptable from a vicarious point of view in Study 1. Role-related concerns were also higher in Study 2 as a result of third-party bias cues, and partly explained participants’ final (zero-sum) candidate choices. Thus, the results of our studies converge with past findings with non-professional samples [12, 13], and provide experimental evidence to complement past field investigations that could not tease out a process whereby staffing recruiters appeared to sort job candidates in anticipation of client preferences [8].

### Practical implications

To our knowledge, we are the first to document the extent to which hiring professionals may encounter situations conducive to third-party bias accommodation in their careers. Our results paint an alarming picture: Hiring professionals in both Ireland and the U.S. estimated that a sizable proportion of clients issue explicit requests not to hire from certain groups (even when doing so is illegal in both countries), including a variety of social category groups in addition to gender groups, such as those based on race/ethnicity, age, and religion (Study 2). Respondents also estimated that, in the absence of direct requests, a similarly high proportion of hiring professionals spontaneously infer client biases. These findings illustrate how perspective taking can sometimes have negative consequences in organizations [67]. In our case, considering client preferences—which many in our sample reported doing—can lead to spreading their biases. The results of Study 1 are particularly noteworthy, as those who perceived bias accommodation as more commonplace also found a specific instance of it to be more acceptable. Thus, although having multiple individuals involved in the hiring process is often recommended to reduce bias [62, 63], the present research shows how professionals who prioritize candidate fit with others accommodate bias and, as observers, condone others’ decisions to do the same.

Channeling others’ biases in hiring decisions runs counter to organizational integrity and may negatively impact the reputation of the HR function itself. The goal or aspiration to build an ethical organizational culture, or to increase diversity and inclusion in the workforce, could be imperiled by the tendency of individual HR professionals to pass along others’ biases when making staff decisions. One way to harness the insights of this investigation for intervention purposes would entail redefining the demands of hiring roles such that rejecting and denouncing bias becomes a central part of the job rather than a discretionary behavior that might compromise role duties [22, 23]. Although, in our studies, the perceived strength of diversity efforts in participants’ own organizations did not relate to participant responses to the hypothetical scenarios (as reported in the S2 Supplement in S1 File), interventions that place those responsibilities on HR professionals within the organization and successfully influence the priority that these professionals give to this goal (among other role demands), might help counter the influence of third-party bias inferences. A strong diversity mission in the organization that the new hire will enter may lead hiring managers to construe their role in a way that centers less on maximizing the fit between potential job candidates and existing organizational members [25] and more on diversifying the organization.

### Strengths, limitations, and remaining questions

The current investigation supports the generalizability of the third-party bias effect from laypersons [12, 13] to trained hiring professionals across a variety of organizations, industries, and ranks. In Study 1, we shed light on how hiring managers might respond to a colleague who engaged in the accommodation of bias against women—an underexamined question that contributes to the further development of role theory and has practical implications for multi-person hiring teams [31]. In Study 2, an experiment, we were able to draw causal inferences on the impact of third-party gender bias on professionals’ own hiring decisions, complementing the findings of prior correlational research [8]. Moreover, asking HR professionals about their own experiences with third-party bias accommodation allowed us to gather evidence for the first time, to our knowledge, suggesting that the phenomenon might apply not only in the context of hiring discrimination based on gender, but also other group contexts (e.g., employment discrimination based on race/ethnicity).

In addition to these strengths, our research also has some limitations that are worth noting. First, we only tested the bias accommodation effect from the actor’s perspective among U.S. participants (in Study 2). Our results suggest that participants in Ireland and the U.S. have similar professional experience with third-party bias (in terms of both explicit requests from clients as well as inferred client preferences to avoid hiring candidates from certain groups). However, it is possible that professionals in Ireland might be even more prone to accommodating bias, given that Irish culture is somewhat more collectivistic than American culture [68]. Hiring professionals in more collectivistic or interdependent cultures [69, 70], which emphasize social cohesion and embeddedness [69, 71], might believe even more strongly in prioritizing fit between existing group members and a newcomer; thus, they might accommodate bias even more readily. Second, we asked participants in Study 2 to make a unilateral hiring decision—a situation that is not representative of all employment selection processes [32]. These findings might not extend to multi-person teams, which sometimes arrive at less biased decisions depending on their composition [31]. Additional research could investigate how role construal at the individual and group level influences responses to third-party bias. Third, whereas we focused on hiring decisions, it may be more common for intermediaries to be tasked with shortlisting candidates; future research may evaluate how third-party bias may influence this shortlisting process based on role construal. Fourth and final, whereas we gathered preliminary evidence in Study 2 indicating that HR professionals have experience with the phenomenon of third-party bias accommodation in the context of groups other than gender groups, future investigations might directly test the accommodation effect in those alternative intergroup contexts. There is some preliminary evidence for the phenomenon in the context of novel groups [72] of which participants had no prior knowledge, stereotypes, or attitudes [13]. Further research is necessary to extend and generalize the third-party bias effect to employment discrimination more broadly.

## Conclusions

A focus on the demands of the roles that people occupy can advance our understanding of how bias spreads in organizations. This investigation reveals that viewing the hiring manager role in a particular way (i.e., endorsing a role demand to maximize the fit between a job candidate and existing organizational members) is associated with more tolerance of bias accommodation among HR professionals, both on the part of the self and another person. The results from the two studies indicate that many hiring professionals indeed may view the act of channeling others’ biases in staff selection as “part of the job,” a behavior most other professionals would engage in and, therefore, acceptable regardless of their personal attitudes. Whereas HR professionals might be expected to minimize bias in recruitment [19], the current studies illustrate how a focus on candidate fit with others promotes unfair hiring practices that lead to employment discrimination on the basis of gender (and potentially other demographic characteristics such as race/ethnicity or age). Thus, a strong focus on hiring based on candidate fit with others may endanger the strides that have been made in reducing gender inequality, and generally run counter to diversity efforts in organizations.

## Supporting information

S1 File (ZIP)
