# Supplementary material for: Individual variation in role construal predicts responses to third-party biases in hiring contexts
Source: PLoS One. 2021 Feb 3;16(2):e0244393. doi: 10.1371/journal.pone.0244393 (PMC7857582; doi:10.1371/journal.pone.0244393)
Supplement: S1 File — (ZIP) [file pone.0244393.s001.zip › S1 Appendix.docx]

**S1 Appendix. Study 1 vignette.**

“Imagine a hiring manager working in personnel selection and placement in a new mid-size company currently looking to recruit a Vice president of operations. The VP of operations will be in charge of designing a winning strategy for the company and oversee the directors and managers as they carry out the plan. The VP of operations will report directly to the company CEO, John A. He is 50 years old, he studied engineering at MIT, and he has been in the field of information technology for the past 12 years. He is married with four children. His wife gave up her job right before their first child was born and has been a stay-at-home mom since. Through the grapevine, the hiring manager has come to learn that the CEO, John A., is known for his politically conservative beliefs as well as his traditional views. John believes that it is important for women to put families before careers and that, given the shortage of openings in today's job market, positions with the most career promise should be given to individuals who are less likely to be distracted by their family life. The last 20 employees that the company selected for a variety of roles have been 80% men and 20% women.

The hiring manager in charge of selecting a new VP of operations found two highly qualified candidates for this position, Brian N. and Karen R. Brian N. and Karen R were comparable in terms of personality traits and aptitudes. They also had comparable education, and similar professional experience in the technology industry. Given the CEO’s conservative views, the hiring manager decided to select Brian N. as new VP of operations.”
