# Supplementary material for: Individual variation in role construal predicts responses to third-party biases in hiring contexts
Source: PLoS One. 2021 Feb 3;16(2):e0244393. doi: 10.1371/journal.pone.0244393 (PMC7857582; doi:10.1371/journal.pone.0244393)
Supplement: S1 File — (ZIP) [file pone.0244393.s001.zip › S1 Supplement.docx]

**S1 Supplement. Study 1 Supporting Information.**

**Table of contents**

Recruitment strategy in Study 12

Power analysis and sensitivity analysis in Study 13

Results adjusting for social desirability scores in Study 14

Skewness analysis in Study 15

S1 supplement references7

**Recruitment strategy in Study 1**

We recruited participants simultaneously in five different ways for Study 1. (As reported in the main text, recruitment source did not impact the study’s results.) One way that we recruited participants for Study 1 was by sharing a link to the study with the professional HR networks of the second and third authors (e.g. via LinkedIn.com); we recruited 78 participants in this manner (24.4% of the sample). Our second recruitment strategy involved partnering with professional organizations to share a link to our survey with HR manager members (*n* = 32; 10% of the sample). A third way was by reaching out to university HR employees (*n* = 14; 4.4% of the sample). A fourth way was by inviting alumni from an HR Master’s program in Ireland to participate (*n* = 92, 28.7% of the sample). In order to ensure a sufficiently large sample, we also invited current students in this program (*n* = 69; 21.6% of the sample). As reported in the main text, there were no systemic effects associated with participant status (HR professional or HR student). Finally, as in other investigations with HR professionals [1], we used a chain referral approach [2] and recruited 31 new participants (10.6% of the sample) by asking existing participants at the end of the study to share our survey with other HR professionals. We were unable to establish recruitment source for 11 participants (3.4% of the sample) due to technical problems.

**Power analysis and sensitivity power analysis in Study 1**

A priori power analyses conducted with G*Power 3 software [3] indicated that we needed *n* = 194 to be able to detect significant correlations of about *r* = .20 with power = .80 and alpha = .05. We increased the target sample size by 20% to make up for missing data or inattention; thus, we aimed to sample at least *n* = 233 participants. For each measure, sample sizes ranged from *n* = 208 to *n* = 271 (i.e., due to missing data in some measures but not others). A sensitivity power analysis using G*Power 3.1 [3, 4] revealed that this was sufficient to detect small effects (*f*^2^ = .05-.06) in a multiple regression with up to five predictors with α = .05 and power = .80.

**Results adjusting for social desirability scores in Study 1**

The social desirability scale was unreliable in our sample; thus, the results reported in the main text do not include this measure. Here, we report ancillary results testing the perceived acceptability of prejudice accommodation as a function of individual difference factors and role demand endorsement, adjusting for social desirability scores. As the regression model reported in the main text, this model included participant gender (-1 = male, 1 = female), SDO scores (mean-centered), the role demand composite (mean-centered), and the two-way interactions between role demand endorsement and participant gender and between role demand endorsement and SDO, as well as social desirability scores (mean-centered) as a covariate. Similar to the model without this covariate, results revealed a significant, positive coefficient for role demand endorsement, *b* = .26, *SE* = .08, *p* = .001, β = .24, and a significant, positive coefficient for SDO, *b* = .13, *SE* = .05, *p* = .014, β = .16. The coefficient for participant gender was no longer significant when social desirability scores were accounted for, *b* = -.11, *SE* = .06, *p* = .073, β = -.12. There were no significant interactions, *p*s > .70.

**Skewness analysis in Study 1**

|  | ***N*** | **Mean** | ***SD*** | **Skew** | ***SE*** |
| --- | --- | --- | --- | --- | --- |
| ***Perceived Acceptability*** |  |  |  |  |  |
| accept1_1: The hiring manager made the right decision. | 255 | 2.03 | 1.015 | 0.779 | 0.153 |
| accept1_2: The hiring manager’s decision was justified. | 255 | 1.97 | 1.083 | 0.907 | 0.153 |
| accept1_3: The decision that the hiring manager made was acceptable. | 255 | 1.99 | 1.079 | 0.943 | 0.153 |
| accept2_1: The hiring manager acted based on what was reasonable. | 252 | 1.91 | 1.068 | 0.928 | 0.153 |
| accept2_2: The hiring manager took a pragmatic stand. | 252 | 2.69 | 1.306 | -0.049 | 0.153 |
| accept2_3: The hiring manager did what was rational to do. | 252 | 2.33 | 1.223 | 0.358 | 0.153 |
| accept2_4: The hiring manager did what was best for the company. | 252 | 1.84 | 0.950 | 0.894 | 0.153 |
| ***Perceived Normativity*** |  |  |  |  |  |
| PREV1_1: Most hiring managers would act in a similar way as the hiring manager in the story. | 246 | 3.11 | 1.194 | -0.206 | 0.155 |
| PREV1_2: The situation described in the story is common. | 246 | 3.48 | 1.149 | -0.599 | 0.155 |
| PREV1_3: The majority of human resources professionals would make the same decision as the hiring manager in the story. | 246 | 2.55 | 1.190 | 0.277 | 0.155 |
| ***Role-related concerns: Interpersonal*** |  |  |  |  |  |
| CONS_I1: The CEO would be displeased working with Karen R. | 240 | 3.14 | 0.880 | -0.095 | 0.157 |
| CONS_I2_r: Karen R. and the CEO would get along together very well (R) | 240 | 3.0458 | 0.75016 | -0.075 | 0.157 |
| CONS_I3: The CEO would not respect Karen R. as VP of operations. | 240 | 3.13 | 1.000 | -0.363 | 0.157 |
| ***Role-related concerns: Task-Focused*** |  |  |  |  |  |
| CONS_P1_r: Karen R. would be a successful VP of operations (R) | 238 | 2.4244 | 0.82712 | -0.298 | 0.158 |
| CONS_P2: Karen R. would have difficulties performing at a high level. | 238 | 2.25 | 1.138 | 0.324 | 0.158 |
| CONS_P3: The company’s performance would suffer. | 238 | 1.97 | 0.989 | 0.578 | 0.158 |
| ***Role-related concerns: Professional*** |  |  |  |  |  |
| CONS_S1: The hiring manager’s credibility in the company might suffer. | 238 | 2.76 | 1.134 | -0.104 | 0.158 |
| CONS_S2: Other people in the company might think that the hiring manager does not care enough about the company. | 238 | 2.26 | 1.087 | 0.581 | 0.158 |
| CONS_S3: The hiring manager might get demoted or fired. | 238 | 2.34 | 1.062 | 0.350 | 0.158 |
| CONS_S4: The hiring manager might be treated less favorably by the CEO in the future. | 238 | 3.46 | 0.970 | -0.803 | 0.158 |
| ***Role demand to prioritize candidate fit with others (GENERAL)*** | |  |  |  |  |
| RE2_1: Recruiters/hiring managers must take into account the preferences of existing company members when making hiring recommendations. | 261 | 3.95 | 1.913 | -0.152 | 0.151 |
| RE2_2: When evaluating job candidates for a new position, anticipating the preferences of existing company members should be a top priority for recruiters / hiring managers. | 263 | 3.48 | 1.882 | 0.226 | 0.150 |
| RE2_3: It is central to the recruiter’s / hiring manager’s role to consider the preferences of existing company employees when hiring new employees. | 268 | 3.74 | 1.902 | -0.019 | 0.149 |
| RE1_S1: When evaluating candidates for a new position, recruiters/hiring managers must take into consideration whether the supervisor’s values provide a good fit with the candidate’s values. | 264 | 3.03 | 1.179 | -0.353 | 0.150 |
| RE1_S2: It is central to the recruiter’s/hiring manager’s role to identify the kinds of candidates whose personal values match those of the supervisor. | 265 | 2.59 | 1.215 | 0.172 | 0.150 |
| RE1_S3: The recruiter’s/hiring manager is responsible for finding candidates whose values are very similar to the values of the supervisor. | 264 | 2.75 | 1.172 | 0.004 | 0.150 |
| RE1_C1: When evaluating candidates for a new position, recruiters/hiring managers must take into consideration whether a candidate’s values provide a good fit with the values of co-workers. | 265 | 3.48 | 1.142 | -0.695 | 0.150 |
| RE1_C2: It is central to the recruiter’s/hiring manager’s role to identify the kinds of candidates whose personal values match those of co-workers. | 264 | 2.98 | 1.166 | -0.144 | 0.150 |
| RE1_C3: The recruiter’s/hiring manager is responsible for finding candidates whose values are very similar to the values of co-workers. | 266 | 3.07 | 1.199 | -0.118 | 0.149 |
| ***Role demand preson-organization fit*** |  |  |  |  |  |
| RE1_O1: When evaluating candidates for a new position, recruiters/hiring managers must take into consideration whether the organization’s values and culture provide a good fit with the candidate’s personal values. | 264 | 4.31 | 0.861 | -1.700 | 0.150 |
| RE1_O2: It is central to the recruiter’s/hiring manager’s role to identify the kinds of candidates whose personal values match those of the organization. | 263 | 4.16 | 0.985 | -1.518 | 0.150 |
| RE1_O3: The recruiter’s/hiring manager is responsible for finding candidates whose values are very similar to the values of the organization. | 265 | 4.28 | 0.927 | -1.581 | 0.150 |

**S1 supplement references**

1. Gorman CA, Meriac JP, Roch SG, Ray JL, Gamble JS. An exploratory study of current performance management practices: Human resource executives’ perspectives. Int J of Sel and Assess. 2017 Jun;25(2):193-202.
2. Bagheri A, Saadati M. Exploring the effectiveness of chain referral methods in sampling hidden populations. Indian Journal of Science and Technology. 2015 Nov;8(30):1-8.
3. Faul F, Erdfelder E, Lang AG, Buchner A. G* Power 3: A flexible statistical power analysis program for the social, behavioral, and biomedical sciences. Behav Res Methods. 2007 May 1;39(2):175-91
4. Faul F, Erdfelder E, Buchner A, Lang AG. Statistical power analyses using G* Power 3.1: Tests for correlation and regression analyses. Behav Res Methods. 2009 Nov 1;41(4):1149-60.
