# Supplementary material for: Individual variation in role construal predicts responses to third-party biases in hiring contexts
Source: PLoS One. 2021 Feb 3;16(2):e0244393. doi: 10.1371/journal.pone.0244393 (PMC7857582; doi:10.1371/journal.pone.0244393)
Supplement: S1 File — (ZIP) [file pone.0244393.s001.zip › S1 Table.docx]

**S1 Table. Explicit Client Requests and Inferred Client Preference to Avoid Hiring Candidates from Various Groups in Study 2.**

| **Social category** |  | Explicit client requests to avoid hiring (*n*=121) |  | Inferred client preference to avoid hiring (*n*=133) |
| --- | --- | --- | --- | --- |
| *Gender-based* |  | *73 (60.3%)* |  | *86 (64.7%)* |
| Female candidates |  | 46 (63.0%) |  | 50 (58.1%) |
| Male candidates |  | 26 (35.6%) |  | 28 (32.6%) |
| Transgender candidates |  | 1 (1.4%) |  | 8 (9.3%) |
| *Race/ethnicity-based* |  | *40 (33.0%)* |  | *55 (41.3%)* |
| White candidates |  | 14 (35.0%) |  | 13 (23.6%) |
| Black/African-American candidates |  | 12 (30.0%) |  | 16 (29.0%) |
| Asian candidates |  | 6 (15.0%) |  | 10 (18.2%) |
| Arab candidates |  | 5 (12.5%) |  | 11 (20.1%) |
| Hispanic candidates |  | 3 (7.5%) |  | 5 (9.1%) |
| *Age-based* |  | *24 (19.8%)* |  | *15 (11.3%)* |
| *Religion-based* |  | *7 (5.8%)* |  | *14 (10.5%)* |
| *Sexual orientation-based* |  | *1 (0.8%)* |  | *7 (5.26%)* |
| *"Other" (e.g., disability)* |  | *42 (34.7%)* |  | *33 (24.8%)* |

*Note:* Participants could select multiple groups; therefore, percentages across different social categories do not add up to 100% within each column.
