# Supplementary material for: Individual variation in role construal predicts responses to third-party biases in hiring contexts
Source: PLoS One. 2021 Feb 3;16(2):e0244393. doi: 10.1371/journal.pone.0244393 (PMC7857582; doi:10.1371/journal.pone.0244393)
Supplement: S1 File — (ZIP) [file pone.0244393.s001.zip › S2 Appendix.docx]

**S2 Appendix B. Study 2 Vignette and Third-Party Gender Bias Cues Manipulation.**

**Study 2 vignette**

“Imagine that you work at a recruitment agency dedicated to personnel selection and placement. A new, mid-sized technology company has recently hired your agency to assist them in the recruitment of a VP of operations. As part of the role, the VP of operations will design a winning strategy for the company and oversee the directors and managers as they carry out the plan. As the new tech company is just starting out, the future of the organization will depend on whether the VP of operations succeeds. Also, your ability to keep your job depends critically on the success of the VP you select. If the VP you select is a failure, your agency's reputation will suffer, and you may lose your job. The VP of operations will report directly to the company CEO.”

**Study 2 third-party bias cues manipulation**

All participants read the following information; the text in italics was only presented to participants in the third-party bias cues condition:

“The CEO, Gary A. is 50 years old *and known for his politically conservative beliefs as well as his traditional views*. Gary studied engineering at MIT and has been in the field of information technology for the past 12 years. *The last 20 employees his company selected for a variety of roles have been 80% men and 20% women*. He is married with four children. *Gary believes that it is important for women to put families before careers and that, given the shortage of openings in today's job market, positions with the most career promise should be given to individuals who are less likely to be distracted by their family life.* His wife gave up her job right before their first child was born and has been a stay-at-home mom since.”
