# Supplementary material for: Individual variation in role construal predicts responses to third-party biases in hiring contexts
Source: PLoS One. 2021 Feb 3;16(2):e0244393. doi: 10.1371/journal.pone.0244393 (PMC7857582; doi:10.1371/journal.pone.0244393)
Supplement: S1 File — (ZIP) [file pone.0244393.s001.zip › S2 Supplement.docx]

**S2 Supplement. Study 2 Supporting Information.**

**Table of contents**

Recruitment strategy in Study 22

Power analysis and sensitivity analysis in Study 23

Effect of experimental manipulation on estimates of explicit client requests in Study 24

Effect of experimental manipulation on estimates of inferred client preferences in Study 25

Results adjusting for social desirability scores in Study 26

Moderation by strength of diversity efforts in participants’ organizations in Study 210

S2 supplement references12

**Recruitment strategy in Study 2**

We employed a three-pronged recruitment strategy in Study 2. (As reported in the main text, recruitment source did not impact the study’s results.) One way that we recruited participants in Study 2 was by partnering with HR practitioners in the U.S. who shared a link to our study with their networks (we recruited 54 participants in this way, 18.4% of the total study sample). Another way was by purchasing verified contact information for 18,842 HR professionals in different kinds of positions within a variety of company types and sizes across the U.S. We sent an email invitation to these contacts—4,009 emails bounced back; the 14,833 delivered emails resulted in 208 participants (71.0% of the total study sample). Finally, as in Study 1, we used a chain referral approach [1, 2], asking participants at the end of the study to share our survey with their fellow HR professionals; 31 participants were recruited in this way (10.6% of the sample).

**Power analysis and sensitivity power analysis in Study 2**

We conducted a priori power analyses with G*Power 3 software [3] to determine the sample size necessary based on the study’s 2 (prejudice cues condition: control vs. prejudice cues) x 2 (participant gender: male vs. female) between-subjects experimental design. Analysis revealed that we needed at least *n* = 199 to have power = .80 (assuming alpha = .05) to detect main effects and a two-way interaction of size *d*=.40, including up to 5 covariates (e.g., social desirability) and 3 counterbalancing variables (see procedure section). Although the internal meta-analysis by Vial, Brescoll, and Dovidio (2019) based on Mturk samples revealed an average main effect of *d*=.55 using similar experimental methodologies, we assumed a more conservative estimate for our professional HR sample. We also increased the target sample size by 10% to make up for missing data or failed attention checks. In total, we aimed to sample at least *n*=220 participants in order to be able to draw meaningful conclusions from the experimental part of the study. As we also wanted to test the moderating effect of role demand endorsement, and given that the second part of the study is aimed at documenting the prevalence of the phenomenon, we aimed to recruit as many participants as it was possible within our budgetary means. After data collection, we employed G*Power 3.1 [3] to conduct a sensitivity power analysis to estimate the minimum effect size that could be detected. For the manipulation check analysis described in the main text, which entailed a 2×2 Analysis of Variance (ANOVA), the analyses revealed that *n* = 246 was sufficient to detect a small-to-medium effect (*d* = .36) with α = .05 and power = .80. Moreover, a regression model (described in the main text) with *n* = 245 and up to 11 predictors [3, 4] was sufficient to detect a medium effect (*d* = .44) with α = .05 and power = .80.

**Effect of experimental manipulation on estimates of explicit client requests in Study 2**

We examined whether the prejudice cues manipulation had any effect on estimates of the prevalence of explicit client requests to avoid hiring candidates from specific groups. A 2 (prejudice cues condition: third-party prejudice cues vs. no cues) *×* 2 (participant gender: male vs. female) ANOVA revealed a non-significant effect of prejudice cues condition, *F*(1, 241) = 1.11, *p* = .292, η*_p_*^2^ = .005. No other effects were significant, *p*s > .288.

**Effect of experimental manipulation on estimates of inferred client preferences in Study 2**

We examined whether the prejudice cues manipulation had any effect on perceptions of the prevalence of HR professionals’ inferences about client preferences to avoid hiring candidates from specific groups. A 2 (prejudice cues condition: third-party prejudice cues vs. no cues) *×* 2 (participant gender: male vs. female) ANOVA revealed a non-significant effect of prejudice cues condition, *F*(1, 242) = 2.53, *p* = .113, η*_p_*^2^ = .010. No other effects were significant, *p*s > .471.

**Results adjusting for social desirability scores in Study 2**

The social desirability scale was unreliable in our sample; thus, the results reported in the main text do not include this measure. Here, we report the results of these analyses adjusting for social desirability.

**Prejudice cues manipulation check, adjusting for social desirability**

A 2 (prejudice cues condition: third-party prejudice cues vs. no cues) *×* 2 (participant gender: male vs. female) Analysis of Co-Variance (ANCOVA) with social desirability scores as a covariate revealed a significant main effect of prejudice cues condition on participants’ perceptions that the third party was biased against working women, *F*(1, 241) = 168.04, *p* < .001, η*_p_*^2^ = .411. There was also a significant main effect of participant gender, *F*(1, 241) = 6.44, *p* = .012, η*_p_*^2^ = .026, as in the analysis reported in the main-text. The interaction was not significant, *F*(1, 241) = 2.72, *p* = .100, η*_p_*^2^ = .011.

**Candidate preferences, adjusting for social desirability**

We conducted regression analysis on the continuous measure of preference for a female candidate over a male candidate, including social desirability scores as a covariate, as well as prejudice cues condition (-1 = no cues, 1 = third-party prejudice cues), participant gender (-1 = male, 1 = female), role demand endorsement (mean-centered), and all interaction terms as predictors of each of the two outcome variables. We also included the three counterbalancing variables described in the procedure as covariate factors. This regression model revealed a non-significant main effect of experimental condition, *b* = -.05, *SE* = .16, *p* = .750, β = -.02, and a significant two-way condition *×* role demand interaction, *b* = -.26, *SE* = .10, *p* = .008, β = -.19. Consistent with Hypothesis 1c, when third-party prejudice cues were present, participants who reported stronger role demand endorsement had significantly lower preference for a female candidate, *b* = -.47, *SE* = .14, *p* = .001, β = -.36. But when prejudice cues were absent, role demand endorsement was not significantly related to participant relative candidate preferences, *b* = .04, *SE* = .13, *p* = .74, β = .03. A significant three-way, condition *×* role demand *×* participant gender interaction also emerged, *b* = .25, *SE* = .10, *p* = .011, β = .18. As in the main text, we decomposed this three-way interaction by probing the responses of male HR professionals and female HR professionals separately.

For male HR professionals, there was a significant two-way condition *×* role demand interaction, *b* = -1.01, *SE* = .33, *p* = .002, β = -.52. In the third-party prejudice cues condition, male participants with stronger role demand endorsement had significantly lower preference for the female candidate, *b* = -.71, *SE* = .24, *p* = .003, β = -.53. In the control condition, male participants with stronger role demand endorsement had a slightly higher (nonsignificant) preference for a female candidate, *b* = .31, *SE* = .23, *p* = .178, β = .23. For female HR professionals, the two-way condition *×* role demand interaction was not significant, *b* = -.03, *SE* = .20, *p* = .899, β = -.01. There were also no significant main effects of condition, *b* = -.10, *SE* = .17, *p* = .544, β = -.05; or role demand endorsement, *b* = -.22, *SE* = .14, *p* = .111, β = -.16.

**Final candidate selections, adjusting for social desirability**

We conducted a binary logistic regression analysis predicting final selection of a female candidate (coded as 1) or a male candidate (coded as 0), adjusting for social desirability, with prejudice cues condition (-1 = no cues, 1 = third-party prejudice cues), participant gender (-1 = male, 1 = female), role demand endorsement (mean-centered), and all interaction terms as predictors of each of the two outcome variables. We also included the three counterbalancing variables described in the procedure as covariate factors. The model revealed no significant effect of experimental condition, *b* = -.10, *SE* = .17, *p* = .552. There was the anticipated significant two-way condition × role demand interaction, *b* = -.32, *SE* = .12, *p* = .006: When third-party prejudice cues were present, participants who reported stronger role demand endorsement were significantly less likely to select the female candidate, *b* = -.65, *SE* = .19, *p* = .001, OR = .523, 95% CI [.357, .766]. When prejudice cues were absent, role demand endorsement was unrelated to participant preference for a female over a male candidate, *b* = -.008, *SE* = .12, *p* = .949. The two-way condition × role demand interaction in the binary logistic regression model adjusting for social desirability scores was qualified by a significant three-way interaction with participant gender, *b* = .26, *SE* = .11, *p* = .026, OR = 1.292, 95% CI [1.032, 1.619]. To decompose this interaction, we examined male and female participants separately.

For male participants, the two-way condition *×* role demand interaction was significant, *b* = -.58, *SE* = .21, *p* = .006: Male participants in the prejudice cues condition were significantly less likely to select a female candidate the more they endorsed the role demand to prioritize candidate fit with others, *b* = -1.11, *SE* = .36, *p* = .002, OR = .331, 95% CI [.162, .674]. In the control condition, male participants with stronger role demand endorsement were slightly (but not significantly) more likely to select the female candidate, *b* = .05, *SE* = .21, *p* = .825.

For female participants, there two-way condition *×* role demand interaction was not significant, *b*=-.06, *SE* = .10, *p* = .509. Instead, there was a significant main effect of condition: Female participants were less likely to select a female candidate when there were cues to third-party prejudice (vs. no cues), *b*=-.33, *SE* = .16, *p* = .039, OR = .717, 95% CI [.523, .983].

**Role-related concerns, adjusting for social desirability**

We tested the same regression model twice, including social desirability scores as a covariate, first on (a) interpersonal concerns, and then on (b) task-focused concerns, with condition (-1 = control, 1 = prejudice cues), participant gender (-1 = male, 1 = female), and their interaction. These models also included the three counterbalancing variables described in the procedure as covariate factors.

First, for interpersonal concerns, the model revealed as expected a significant effect of condition, such that interpersonal concerns about hiring a female candidate were higher in the third-party prejudice cues condition (vs. control), *b* = .42, *SE* = .05, *p* < .001, β = .50. There was also a main effect of participant gender, such that female HR professionals had overall stronger interpersonal concerns regardless of experimental condition, *b* = .12, *SE* = .05, *p* = .024, β = .13, but the interaction between the two predictors was not significant, *p* = .575.

Second, for task-focused concerns, the model similarly revealed a significant effect of condition, *b* = .17, *SE* = .06, *p* = .008, β = .20: As anticipated, task-focused concerns about hiring a female candidate were higher in the third-party prejudice cues condition (vs. control). No other effects were significant, *p*s > .566.

**Moderation by strength of diversity efforts in participants’ organizations**

We examined whether the strength of diversity efforts in participants’ own organizations was related to their responses to the hypothetical hiring scenario in Study 2. In order to do this, we conducted (a) regression analysis on the continuous measure of preference for a female candidate over a male candidate, and (b) binary logistic regression analysis to examine participant’s final candidate selection (i.e., male candidate vs. female candidate). In both models reported in this supplement, we included prejudice cues condition (-1 = no cues, 1 = third-party prejudice cues), participant gender (-1 = male, 1 = female), strength of diversity efforts in participants’ own organizations (mean-centered), and all interaction terms as predictors of each of the two outcome variables. We also included the three counterbalancing variables described in the procedure as covariate factors.

**Preference for a female candidate over a male candidate, moderation by diversity efforts**

The first regression model revealed a non-significant coefficient for strength of diversity efforts, *b* = .15, *SE* = .10, *p* = .154, β = .15, and a non-significant interaction between experimental condition and strength of diversity efforts, *b* = -.14, *SE* = .11, *p* = .178, β = -.14. Thus, self-reported strength of diversity initiatives in participants’ own organizations did not moderate participants’ relative preference to hire a female over a male candidate as a function of prejudice cues condition.

**Final selection of a female versus a male candidate, moderation by diversity efforts**

The binary logistic regression model revealed a non-significant coefficient for strength of diversity efforts, *b* = .16, *SE* = .11, *p* = .144, OR = 1.176, and a non-significant interaction between experimental condition and strength of diversity efforts, *b* = -.01, *SE* = .11, *p* = .993, OR = .999. Thus, self-reported strength of diversity initiatives in participants’ own organizations did not moderate participants’ final candidate selection as a function of prejudice cues condition.

**S2 supplement references**

1. Gorman CA, Meriac JP, Roch SG, Ray JL, Gamble JS. An exploratory study of current performance management practices: Human resource executives’ perspectives. Int J of Sel and Assess. 2017 Jun;25(2):193-202.
2. Bagheri A, Saadati M. Exploring the effectiveness of chain referral methods in sampling hidden populations. Indian Journal of Science and Technology. 2015 Nov;8(30):1-8.
3. Faul F, Erdfelder E, Lang AG, Buchner A. G* Power 3: A flexible statistical power analysis program for the social, behavioral, and biomedical sciences. Behav Res Methods. 2007 May 1;39(2):175-91
4. Faul F, Erdfelder E, Buchner A, Lang AG. Statistical power analyses using G* Power 3.1: Tests for correlation and regression analyses. Behav Res Methods. 2009 Nov 1;41(4):1149-60.
